# Carbon starvation induces coincident capsule and cell wall remodeling in *Cryptococcus neoformans*

**DOI:** 10.1128/mbio.03701-25

**Published:** 2025-12-30

**Authors:** Elise Bedford, Leandro Buffoni Roque da Silva, Daniel Smith, Christopher W. J. Lee, Quigly Dragotakes, Arturo Casadevall, James W. Kronstad

**Affiliations:** 1 The Michael Smith Laboratories, Department of Microbiology and Immunology, University of British Columbia 8166, Vancouver, British Columbia, Canada; 2 W. Harry Feinstone Department of Molecular Microbiology and Immunology, Johns Hopkins Bloomberg School of Public Health 25802 , Baltimore, Maryland, USA; Instituto Carlos Chagas, Curitiba, Brazil

**Keywords:** glucose, chitin synthase, RNA-seq, capsule permeation, calcofluor white, cell wall, nutrients

## Abstract

The pathogenesis of *Cryptococcus neoformans* is largely attributed to the impact of the polysaccharide capsule on the survival of the fungus in the harsh conditions of the host. These conditions include a robust immune response and nutrient limitation in different tissues. Little is known about the survival mechanisms of *C. neoformans* in nutrient-deprived conditions, and a key unanswered question is whether the fungus can use capsule material for nutrition during conditions of carbon limitation. We addressed this question by measuring alterations in capsule and cell wall in response to carbon limitation and found an influence on capsule porosity and density without a change in diameter. RNA-seq analysis of the response to carbon limitation identified transcripts for enzymes with potential relevance to polysaccharide changes, including carbohydrate-active enzymes. Subsequently, the impact of a selected set of enzymes was evaluated with capsule and cell wall-relevant mutants lacking Cas1 and Cas3 (O-acetylation of capsule polysaccharide), Chs1-8 (chitin synthases), Cps1 (hyaluronic acid synthase), and Kre64 (β-glucan specific glycosidase). Overall, our findings show that *C. neoformans* responds to carbon starvation by increasing capsular and cell wall permeability through interactions between cell wall components (α- and β-glucans and chitin) and capsules that alter their density and porosity. The results also suggest that *C. neoformans* does not substantially degrade the capsule polysaccharide under the conditions of carbon limitation employed in this study.

**IMPORTANCE:**

The World Health Organization recently placed *Cryptococcus neoformans* in the critical priority group of fungal pathogens that threaten human health. The elaboration of a polysaccharide capsule is a major contributor to the ability of *C. neoformans* to cause disease. However, the mechanisms of capsule formation are not well understood, and it is unknown whether the fungus can degrade the polysaccharide upon nutrient limitation. Here, we examined capsule degradation by starving the cells for glucose and monitoring changes in capsule permeability and binding of the dye calcofluor white to the cell wall. We found that permeability and dye binding increased with starvation. A parallel transcriptome analysis revealed candidate functions involved in the response to glucose availability, and subsequent tests with the corresponding mutants indicated an intricate connection between the cell wall and the capsule.

## INTRODUCTION

*Cryptococcus neoformans* is an encapsulated basidiomycetous yeast that causes cryptococcosis and meningoencephalitis in immunocompromised individuals (1). The fungus causes ~220,000 new cases worldwide each year, and cryptococcal meningitis accounts for 19% of acquired immunodeficiency syndrome (AIDS)-related deaths annually (2–4). *C. neoformans* produces a number of key virulence factors including the deposition of melanin pigment in the cell wall, secretion of hydrolytic enzymes, and formation of a large polysaccharide capsule (1, 4). These factors are particularly important for mediating interactions with phagocytic cells. For example, capsule influences uptake by and survival in macrophages, and melanin confers resistance to killing by reactive oxygen species produced by phagocytes (5, 6).

The capsule is considered the main virulence factor of *C. neoformans,* with acapsular mutants generally being avirulent in murine models (5, 6). The mechanisms of capsule elaboration have been intensively studied. The capsule can be induced by a variety of factors, most of which are attributable to the host environment such as low iron, high CO_2_, mammalian serum, mannitol, and nutrient limitation (7–10). Capsule size and composition are influenced by *in vitro* culture conditions, and the cryptococcal species and the cell cycle also have an influence (9, 11). Capsule structure is also influenced by the host, as indicated by cells recovered from the lung, which show larger capsule structures compared with cells from the brain (12). An important feature of capsule growth is that it is a seemingly irreversible process; once a capsule is induced, it appears that it can only grow in size, although the capsule may become smaller during cell aging (13, 14). During capsule growth, there is intermixing of new and old capsular subunits near the edge, and subunits with larger diameters appear to extend beyond the existing capsule structure (14–16).

The growth of capsule and production of capsular components are regulated by a complex network of transcription factors as well as the Pka1-cAMP and MAPK signal transduction pathways (17, 18); these factors and pathways can be activated by low pH, hypoxia, low iron, and low glucose (19, 20). Capsule components are thought to be synthesized in the endoplasmic reticulum (ER) and then exported to the extracellular space in vesicles (21–23). These vesicles then release the polysaccharide fibers that can attach to the cell wall or to existing capsular components (15, 24, 25). Attachment to the cell wall specifically requires α−1,3-glucan (26). In general, the capsule structure is not uniform; the thickness/size of the capsule and internal structure can both vary (27). Moreover, capsule porosity and density vary depending on the environment (12, 13, 28–30). Notably, the integrity of the capsule relies on the stability of and connection to the cell wall (31). The cell wall acts as a barrier for environmental stress, maintains cellular morphology, and contributes to stability and permeability of the plasma membrane (32). The cell wall is composed of two layers of glycans: an outer layer of primarily α- and β-glucans and a cross-linked inner layer of β-glucans and chitin (31–33). Along with glucans, the chitin oligomers present in the cell wall play a role in capsule organization and attachment (27, 33–36).

To investigate capsule remodeling and potential degradation in response to carbon limitation/starvation, we first demonstrated changes in capsule density and porosity by assaying permeability to dextran sulfate and examining binding of calcofluor white to the cell wall. Subsequently, we employed RNA-seq to identify transcriptional changes and generate hypotheses about specific functions involved in the response to carbon limitation relative to glucose reintroduction. A key hypothesis from this analysis was that carbon limitation would provoke changes in transcript levels for enzymes involved in the synthesis of cell wall polysaccharides to influence capsule structure. We tested this idea by using the assays for permeability to examine the influence of loss of enzymes related to capsule synthesis or the cell wall. This analysis revealed that defects in cell wall polysaccharide synthesis impact capsule remodeling during carbon limitation, thus reinforcing the intricate connections between capsule polysaccharide elaboration and the cryptococcal cell wall.

## RESULTS

### Assays for capsule and cell wall remodeling upon carbon limitation

**Fig 1:**
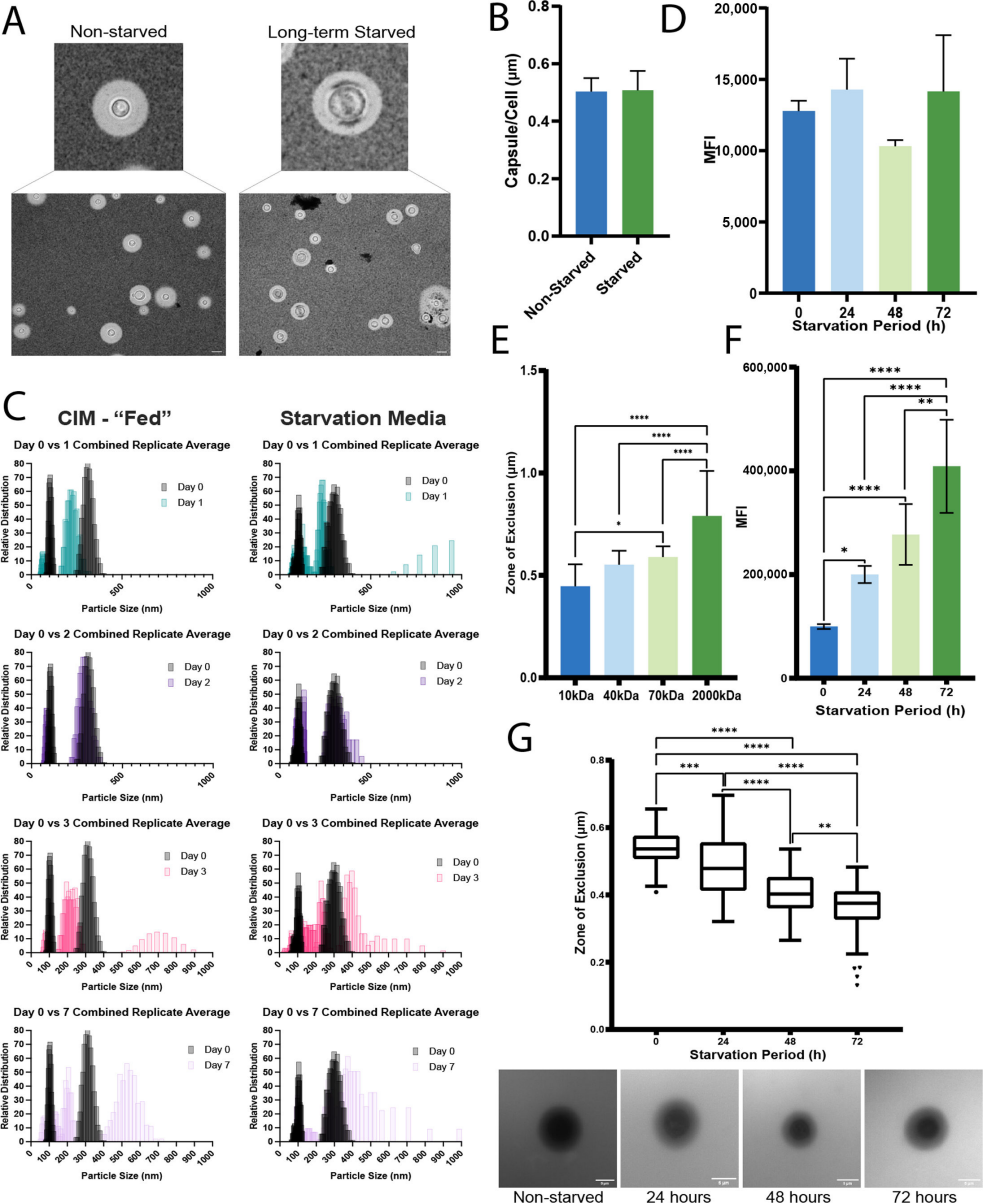
The WT strain H99 shows changes in capsule permeability and cell wall accessibility upon carbon starvation. (**A**) Images of *C. neoformans* cells after 7 weeks in refreshed (non-starved) or unrefreshed (long-term starved) minimal media. (**B**) Capsule thickness was measured before and after 48 h of starvation. The capsule thickness was measured and normalized to cell size with ImageJ. (**C**) Binding pattern of the 18B7 monoclonal antibody to capsule polysaccharide at each time point of starvation, measured using flow cytometry. (**D**) The zone of exclusion for the capsule was measured after 60 min of incubation with the dextrans of the indicated sizes. (**E**) A calcofluor white (CFW) binding assay was used to determine binding intensity as the mean fluorescence intensity (MFI). (**F**) The dextran permeation assay was employed with the 40 kDa dextran and a 60-min incubation to reveal increased permeation as starvation continued. Representative microscopy images below the graph demonstrate the reduction in size of the dark zone of exclusion as starvation continues. (**G**) Dynamic light scattering plots of capsular polysaccharide isolated by sonication. The left column illustrates the “fed” (non-starved) control, and the right column shows the starved samples. Statistical significance was measured with an ordinary one-way ANOVA, and the indicated significance was based on the results of the post-hoc comparisons if the *P*-value was statistically significant in the ANOVA summary (^*^P<0.05, ^**^P<0.01, ^***^P<0.001, ^****^P<0.0001).

Long-standing observations with India ink staining indicate that the *C. neoformans* capsule does not become smaller in diameter during nutrient deprivation and host infection (12, 13, 27). We initially confirmed this observation by determining whether there were differences in India ink staining and capsule diameter pre- and post-carbon limitation. We define carbon limitation/starvation as incubation of the cells in the absence of glucose in minimal medium (see Materials and Methods). The condition of low glucose is relevant to the situation that *C. neoformans* would encounter in the pulmonary environment upon inhalation of yeast cells. That is, the glucose concentration in airway surface liquid is quite low (~0.4 mM) (37). The staining of cells after 7 weeks of carbon limitation revealed patches of India ink within the capsule, suggesting increased permeability (Fig. 1A). Consistent with the literature, measurements of capsule diameter did not reveal differences between cells pre- and post-carbon limitation (Fig. 1B) (12, 13, 27). Additionally, mAb 18B7 staining was performed to determine if the outer capsular structure displayed different binding patterns after a period of starvation; however, no difference in binding was found (Fig. 1C; Fig. S1). We therefore more closely investigated capsule changes upon carbon limitation by (i) implementing two assays of capsule permeability and cell wall staining, (ii) testing growth with cell wall stressors, and (iii) evaluating polysaccharide fibers by scanning electron microscopy (SEM), transmission electron microscopy (TEM), and dynamic light scattering (DLS).

The capsule of *C. neoformans* is not a uniform structure as illustrated, for example, by the examination of permeation by TMR dextrans (12, 28, 30, 36, 38). We initiated an examination of carbon limitation with this permeation assay by incubating encapsulated wild-type (WT) cells with TMR dextrans of 10, 40, 70, or 2,000 kDa. As seen by others, this approach demonstrated that the capsule has a variable structure with a denser matrix near the cell wall and reduced density as the matrix extends from the cell wall (Fig. 1D) (28). We then measured the changes in capsule density during carbon limitation by incubating non-starved cells and cells that were starved for 24, 48, and 72 h with the 40 kDa TMR dextran (Fig. 1F). The cells were first grown in a capsule-inducing medium (CIM; 0.5% glucose) for 48 h and then moved to the same medium without glucose. At the various time points, we measured a “zone of exclusion,” which captured the degree of permeation of the TMR dextran through the capsule (28). The zone was defined as a ratio of the cell plus capsule diameter to the diameter after permeation by the TMR dextran (Fig. S1). This approach accounts for variability in cell and capsule size, and the assay revealed increased permeation during the course of carbon limitation for the WT strain (Fig. 1F).

An additional assay employed flow cytometry to measure changes in the level of calcofluor white (CFW) access and binding to the cell wall upon carbon limitation. Given the small size of CFW, this assay primarily assessed changes in cell wall binding, and the results revealed potential capsule and cell wall alterations for WT cells during carbon limitation (Fig. 1E). These changes were indicated by a significant increase in CFW binding for the starved cells at 24 h (200,028 MFI), 48 h (276,999 MFI), and 72 h (408,606 MFI) relative to the non-starved cells (99,650 MFI) (Fig. 1F). In support of the changes in CFW binding to the cell wall, an increase in sensitivity of the starved cells compared with the non-starved cells was observed in spot assays on solid media containing Congo Red or CFW, shown in Fig. S2. The starved cells showed poor growth, indicating increased sensitivity even after prolonged incubation on the media with glucose, and none of the limitation periods provoked greater sensitivity relative to each other.

To ascertain whether starvation media resulted in structural modifications to the capsular polysaccharide (CPS), we employed ultrasonication to remove fragments of the capsule from cultures grown in either CIM or starvation media and used dynamic light scattering (DLS) to quantify populations of the removed polysaccharide. The initial culture (48 h in CIM with 0.5% glucose) had two CPS populations, one at ~100 nm and another at ~300 nm (Fig. 1G). We found that the size of CPS fragments increased in both of the cultures grown in the CIM with 0.5% glucose and the starvation media over the course of 1 week. However, with the CPS samples collected from starvation conditions, there were additional CPS populations that appeared beyond those previously observed and those identified in the non-starved controls. The appearance of these minor CPS populations may suggest changes in the capsule fibril structures because of transitory rearrangements.

**Fig 2:**
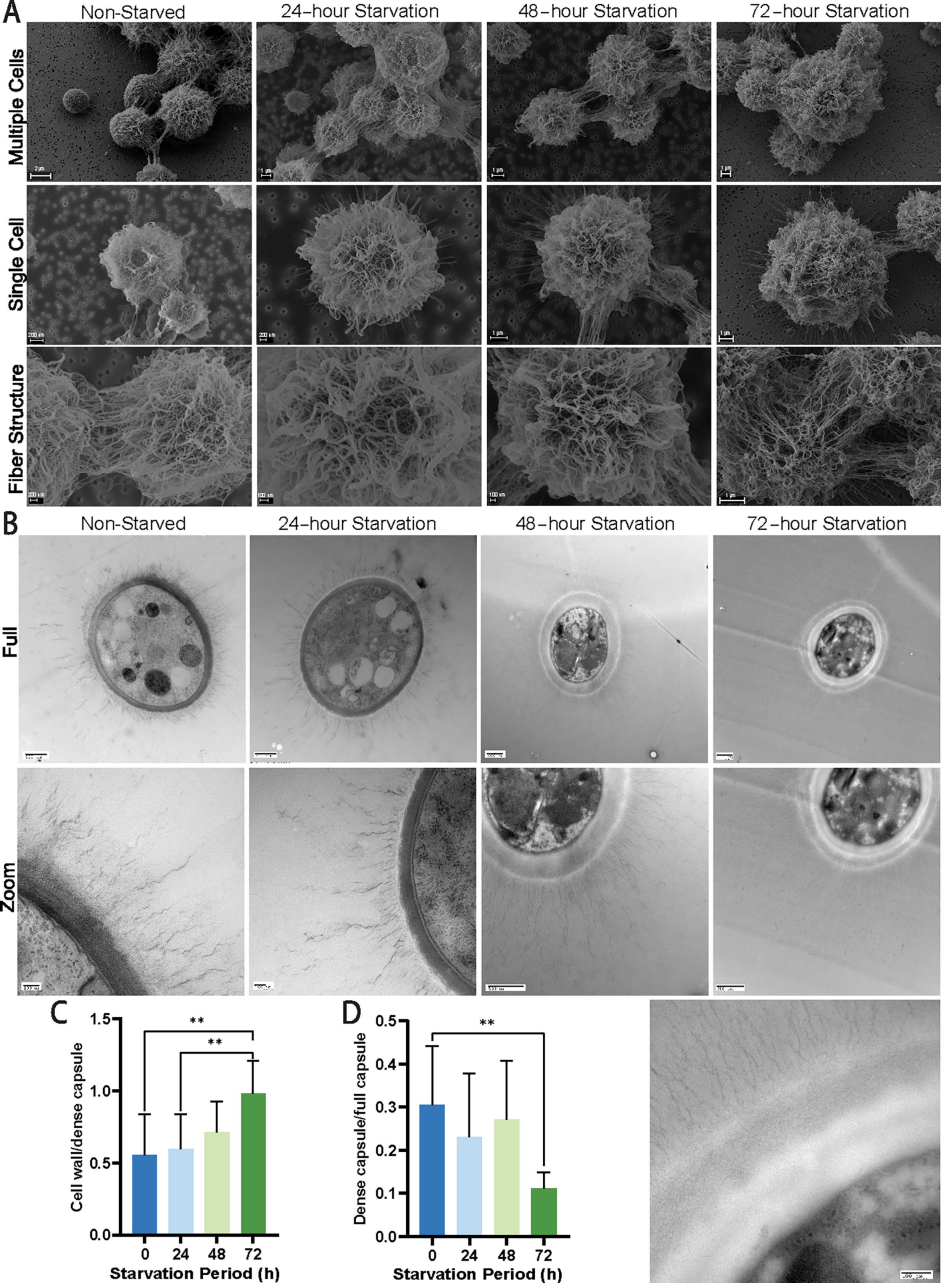
Electron microscopy images of *C. neoformans* capsule before and during carbon starvation. (**A**) SEM images of different magnification to show multiple cells, single cells, and fiber structure on cells from non-starved cultures and cultures of different durations of starvation. The representative images show the polysaccharide fibers in each of the categories. Size bars are shown and range from 100 nM for the fiber images to 2 μM for the multiple cell images. (**B**) TEM images of different magnification showing full cells and zoomed in to show the cell wall and capsule structure at each starvation period. The full cell size bars are 500 nM, and the close-up images are 100 nM for non-starved and 24 h, and 500 nM for 48 and 72 h. (**C**) Ratio between the cell wall size and the dense capsule at each starvation point from the TEM images, which showed an increase over the starvation periods. (**D**) Ratio between the dense capsule and the full capsule, illustrating a downward trend demonstrating that the dense portion of the capsule is decreasing over time.

The conditions for the permeation and CFW assays were also employed with scanning electron microscopy to examine the ultrastructure of capsule fibers upon carbon limitation (Fig. 2A). We found that the non-starved cells displayed more dense and branched polysaccharide fibers compared with the starved cells. Notably, the starved cells from 24 h and 48 h could not be distinguished from one another, while the cells from 72 h were structurally different from any of the other conditions. That is, capsule fibers from cells at 72 h were less branched and appeared “fluffy” compared with the fibers on cells from the other conditions. Higher magnifications further illustrated that the cross-linking of fibers appeared denser for the non-starved cells compared with the 72 h cells. Additional changes for the starved cells included more individually visible fibers and more gaps between the fibers allowing greater observation of deeper capsule layers compared with the non-starved cells. Since these observations were only clearly present by the 72 h period, and our assays had demonstrated additional changes starting at 24 h of carbon starvation, we also used transmission electron microscopy (TEM) to further analyze the cell wall and inner capsular structure.

The TEM analysis, which employed cells from the conditions described above, revealed differences in the cell wall, the inner capsule, and the cell wall-capsule interface between starved and non-starved cells (Fig. 2B through D). The measurements taken from these images included cell wall thickness, dense capsule thickness, and overall capsular thickness (as illustrated in Fig. S1). Using these measurements, ratios were taken between the cell wall size and the dense capsule, which showed an increase over the starvation periods (Fig. 2C), indicating that there was an increase in cell wall thickness and a decrease in capsule density or thickness. The ratio between the dense capsule and the full capsule (Fig. 2D) showed a downward trend demonstrating that the dense portion of the capsule decreased over time. This change can be seen in the representative images as well, where the cell wall showed an increased thickness and the presence of multiple striations (Fig. 2B). We also observed that the dense capsular layer was more difficult to distinguish from the cell wall in comparison to the non-starved or 24 h starved cells. These alternations to thickness and morphology were also seen in the long-term incubated cells, which have a “paler” cell wall compared with the non-starved, glucose cells; this difference was also seen in the TEM images from the starved cells. Overall, these assays support the conclusions that carbon limitation induces remodeling of the capsule resulting in increased permeability and that changes in the cell wall also occur relative to non-starved cells. The duration of carbon limitation appeared to influence the extent of remodeling such that the capsule became less dense with time.

Additional experiments were performed to assess the impact of starvation on viability, recovery in glucose medium, and susceptibility to phagocytosis. We tested the viability of the cells from the carbon limitation experiments by performing propidium iodide (PI) staining and flow cytometry (Fig. S3). This experiment revealed that starving the cells for carbon did not result in a high percentage of dead cells (less than 4% at each time point), and, in fact, a higher level of dead cells was observed for the cells from the non-starved culture (Fig. S3). The ability of the starved cells to recover and resume growth was also tested in rich medium (YPD) and the CIM medium with glucose (Fig. S4). In YPD, the non-starved cells showed the most rapid growth and reached the highest OD, and similar results were found for the 24 h starved cells. The starved cells from the 48 and 72 h cultures showed slower growth and reached a lower density. No obvious differences in growth were observed between any of the cultures in the CIM condition (Fig. S4). Finally, we found that a higher percentage of the cells of a macrophage-like cell line took up the starved versus non-starved yeast cells (Fig. S5). This result is consistent with alterations in the capsule and cell wall to enhance recognition by phagocytic cells.

### Analysis of transcriptome changes during glucose limitation

**Fig 3:**
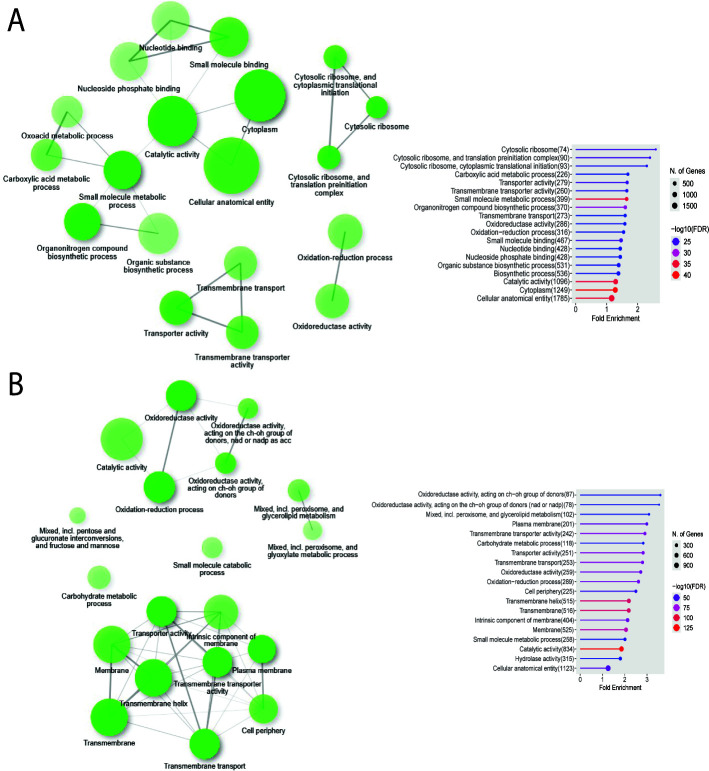
Identification of differentially expressed biological functions between the glucose and starvation conditions by RNA-seq analysis. (**A**) Summary of the functions upregulated in the glucose condition. The network of functions is shown on the left and the number of genes and the fold enrichment of each of the categories are shown on the right. (**B**) Summary of the functions upregulated in the starvation condition. The network of functions is shown on the left, with darker colors representing the number of genes associated with that category, and the number of genes and the fold enrichment of each of the functions are shown on the right. Graphs were created using GO enrichment analysis and include all of the genes with a log2 fold change of one and above and a *P* value<0.05.

We next employed RNA-seq to examine transcriptional responses in cells that were either starved in medium with no glucose or exposed to reintroduced glucose. The goal was to potentially identify differential regulation of enzymes for degradation of the capsule material and functions for recycling capsule sugars. The RNA-seq analysis revealed ~5,000 differentially expressed genes between the condition that had glucose reintroduced (samples labeled “Glu” or Glucose) and the condition of carbon limitation (samples labeled Cap or Starvation) (Tables S1 and S2). Principal component analysis (PCA) indicated a 97% variance among samples representing glucose limitation vs. reintroduction and a 3% variance between replicates. An examination of the gene ontology (GO) terms revealed diverse functions and pathways represented by the differentially expressed genes (Fig. 3A and B). Specifically, the condition of glucose reintroduction identified upregulation of the carbohydrate derivative biosynthetic process, DNA replication, Golgi vesicle transport, mitotic cell division, ribonucleotide binding, cytoskeleton, and ATP binding (Fig. 3A). This pattern is consistent with cell proliferation stimulated by glucose (Fig. 3A). In contrast, analysis of the carbon limitation condition revealed upregulation of genes encoding hydrolase activity (hydrolyzing O-glycosyl compounds), oxidoreductase activity, lipase activity, carbohydrate metabolic processes, acetyl-CoA dehydrogenase activity, and glycerolipid metabolism (Fig. 3B). The regulation of these functions suggested that carbon limitation provoked a remodeling of metabolism and recycling to use alternative carbon sources (e.g., lipids). A heatmap was created to illustrate the differential expression of the known capsule-related genes; these genes were mainly associated with capsule formation and transport (Fig. S6). The majority of the genes in this category were upregulated in the glucose condition, suggesting capsule formation and repair, processes presumed to be absent during carbon starvation (Fig. S6). A second heatmap was generated to display the transcription patterns for genes that followed a similar expression trend to the capsule-related genes, including biosynthesis genes being primarily upregulated in the glucose condition (Fig. S7). As described below, these genes encoded cell wall-related functions that were the targets of further analysis using deletion mutants and the capsule assays illustrated in Fig. 1.

We considered that the RNA-seq data would potentially reveal regulated genes relevant to carbohydrate modification upon carbon limitation. We therefore specifically examined the transcript levels for carbohydrate-active enzymes (CAZymes) present in the top 100 regulated transcripts in the starved versus non-starved data. These data are summarized in a histogram in Fig. S8. This analysis revealed transcripts for three candidate CAZymes upregulated in the carbon limitation condition (Table 1). These included the protein encoded by CNAG_04993 that is predicted to be part of glycoside hydrolase family 115 based on sequence analysis and ortholog characterization. The identity of the protein was determined using an ortholog from *Scheffersomyces stipitis,* which is a hemicellulolytic α-glucuronidase (EC. 3.2.1.131) with a 54% similarity to CNAG_04993 polypeptide (39). Another identified protein, encoded by CNAG_03525, is predicted to belong to the glycoside hydrolase family 37 associated with α,α-trehalases. In particular, the polypeptide encoded by CNAG_03525 has 90% sequence identity with the protein encoded by the Nth2 trehalase enzyme identified in the JEC21 strain of *C. neoformans* (40). Finally, the enzyme encoded by CNAG_00588 has a predicted carbohydrate binding module (CBM). The CNAG_00588 product is part of CBM13, and this group, as well as CBM42, possesses a β-trefoil fold and is part of the CBM fold family 2 (41).

**TABLE 1:** Differentially expressed CAZy enzymes from the top 100 upregulated genes in both conditions^*a*^

Gene	Predicted enzyme	Log2Fold	Condition
CNAG_04993	Glycosyl hydrolase family 115	−5.27	Starvation
CNAG_03525	α,α−trehalase	−6.19	Starvation
CNAG_00588	Ricin-type beta-trefoil lectin domain, CBM family 2	−5.27	Starvation
CNAG_05264	α-amylase, GH family 13	5.35	Glucose
CNAG_04963	Glycosyl hydrolase catalytic domain-containing protein, GH family 128	6.12	Glucose
CNAG_06081	Glucose oxidase, auxiliary activity enzyme AA3	4.01	Glucose
CNAG_00407	Glyoxal oxidase, auxiliary activity enzyme AA5	5.04	Glucose
CNAG_03326	Chitin synthase 2	3.87	Glucose

^*a*^ Negative values represent upregulation in the starvation condition, and positive values represent upregulation in the glucose condition.

The transcripts for five candidate CAZymes were elevated in the non-starved (glucose) condition, and these enzymes are listed in Table 1. Two of these transcripts encoded potential glycoside hydrolases, one from GH family 13 subfamily 1 encoded by CNAG_05264 and one from GH family 128 subgroup VII encoded by CNAG_04963. The GH family 13 enzyme has high sequence similarity to α-amylase, whereas the GH family 128 enzyme may act on β−1,3-glucans. Two other elevated transcripts appeared to encode auxiliary activity enzymes in the AA3 and AA5 families. *C. neoformans* has two enzymes within AA3, but the identified enzyme (CNAG_06081, part of AA3 subfamily 2) was the only one upregulated in the RNA-seq data. The enzyme has sequence similarity to glucose oxidases (42). The upregulated AA5 subfamily gene (CNAG_00407) is highly conserved in Cryptococcus species, and associated reactions for this subgroup involve glyoxal or 2-exopropanol as the donor and molecular oxygen as the receptor to produce glyoxylate or pyruvate and hydrogen peroxide (43). The fifth gene that showed upregulation in the glucose condition (CNAG_03326) encodes chitin synthase 2 (Chs2), which polymerizes chitin via transfer of the sugar moiety of UDP-GlcNAc to the non-reducing end of the growing polymer (44, 45). The loss of Chs2 (among the eight chitin synthase genes in *C. neoformans*) leads to abnormal fiber distribution in the capsule as well as a reduced capsule diameter (45). The observed upregulation may indicate that the enzyme is needed to repair/modify the cell wall when a carbon source is reintroduced.

The discovery of regulation of the chitin synthase gene (Chs2) prompted an examination of regulation of other members of the *CHS* group of genes (Fig. S7, and see below) and, most notably, an examination of regulatory factors for these genes. Ras1 is known to regulate the transcript levels of *CHS2* (46), and our RNA-seq analysis revealed elevated transcripts of the *RAS1* gene (log2 fold = 1.60) and the *RAS2* gene (log2 fold = 1.48) in the glucose condition. An examination of the data also revealed that many of the known components of the Ras pathway were upregulated in the glucose condition, as demonstrated by the *CHS* genes (Table 2).

**TABLE 2:** Trends for each of the mutant strains in the TMR dextran permeation and CFW binding assays, and the log2 fold change of each of the deleted genes is shown from the RNA-seq data^*a*^

Strain	RNA-seq (log2 fold)	Zone of exclusion trend	CFW binding intensity trend
H99	N/A	Linear decrease	Linear increase
*cas1Δ*	3.23	Plateau decrease	Slight increase
*cas3Δ*	3.94	No change	Linear increase
*kre64Δ*	−3.18	No change	Linear increase
*cps1Δ*	2.16	Linear decrease	Increase (high at 48 h)
*chs1Δ*	Not present	Slight decrease	Increase (high at 48 h)
*chs2Δ*	3.87	Increase	Increase (high at 48 h)
*chs3Δ*	0.46	Slight decrease	Decrease from 24 h
*chs4Δ*	−0.68	Decrease	Increase (high at 48 h)
*chs5Δ*	0.98	Slight increase	Increase (high at 24/48 h)
*chs6Δ*	2.5	Slight increase	Increase (high at 24/48 h)
*chs7Δ*	−0.68	Decrease	Increase (high at 48 h)
*chs8Δ*	−0.87	Slight decrease	Decrease from 24 h
*cda1Δ*	0	Increase (high at 24 h)	Linear increase

^*a*^ Positive values represent upregulation in the glucose condition, and negative values represent upregulation in the starvation condition.

### Mutants defective in hyaluronic acid synthesis and capsule O-acetylation have altered capsule permeability

**Fig 4:**
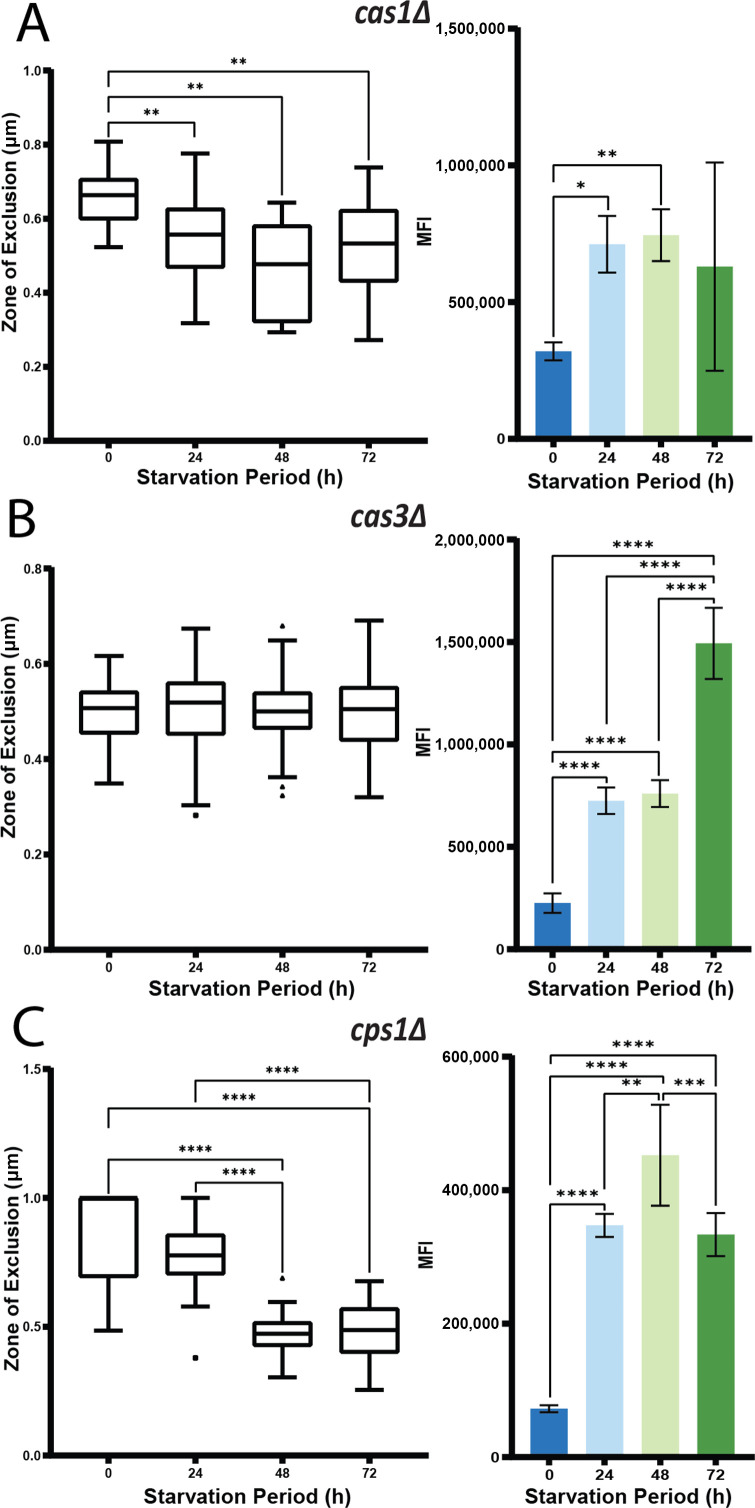
Mutants impaired in O-acetylation and hyaluronic acid display capsule and cell wall alterations during carbon starvation. (**A**) Assay results for (**A**) the *cas1*Δ mutant, (**B**) the *cas3*Δ mutant, and (**C**) the *cps1*Δ mutant with the dextran permeation assay presented on the left and the CFW binding assay results on the right. Statistical significance was measured with an ordinary one-way ANOVA, and the labeled significance was based on the results of the post-hoc comparisons if the p-value was statistically significant in the ANOVA summary (^*^P<0.05, ^**^P<0.01, ^***^P<0.001, ^****^P<0.0001).

The observed regulation of the *CHS2* transcript upon glucose reintroduction prompted a reexamination of the RNA-seq data for the carbon limitation response of transcripts related to the cell wall and capsule functions. We hypothesized that these functions may be critical for capsule remodeling, and this idea is supported by published studies on chitin and hyaluronic acid connections with the capsule (45, 47). To test our hypothesis, we employed the capsule permeability assays to examine mutants in three groups: (i) capsule modification (*cps1*Δ, *cas1*Δ, *cas3Δ,* and *pbx2*Δ); (ii) β−1,6-glucan synthesis (*kre*Δ and *skn1*Δ); and (iii) chitin synthesis/modification (*chs1-8*Δ and *cda1*Δ). Table 2 lists the selected set of genes and their observed regulation in the RNA-seq data (a heatmap analysis is shown in Fig. S7). The transcripts for *CAS1* and *CAS3* were upregulated upon glucose reintroduction, and current evidence suggests that these proteins are involved in O-acetylation of the capsular GXM (48–50). The *cas1Δ* mutant displayed a decrease in permeation of the dextran between non-starved and starved samples that was not as dramatic as with WT cells, and no significant difference was seen between the cells at the different time points (Fig. 4A). The CFW binding assay demonstrated a slight increase in MFI between the non-starved and the 24 and 48 h samples, but no measurable differences were observed between the glucose-limited samples. These results suggest that Cas1 has a minor influence on the capsule response to carbon limitation. Cas3 is one of six Cap64 homologs (Cas3, Cas31, Cas32, Cas33, Cas34, and Cas35) (49). The *cas3*Δ mutant displayed no change across any of the time points with the dextran permeation assay indicating a requirement for Cas3 in the response to carbon limitation (Fig. 4B). The *cas3*Δ mutant followed a similar trend to the WT in the CFW binding assay, with the only difference being a plateau of MFI at 24 and 48 h. These results suggest that the GXM alterations affected by carbon limitation response are influenced by Cas3.

Cps1 synthesizes cell surface hyaluronic acid that contributes to *C. neoformans* virulence (51, 52). Hyaluronan is a high molecular mass (1,000–5,000 kDa) anionic and linear polysaccharide composed of β−1,4-linked repeating disaccharides of glucuronic acid and β−1,3-linked N-acetylglucosamine (52). The *CPS1* transcript was upregulated in the glucose condition (Table 2; Fig. S7), and the dextran permeation markedly increased during carbon starvation of the mutant (Fig. 4C). The CFW binding assay revealed that the starved cells had a significantly higher MFI value at 24 h compared with the non-starved cells, with a high level at 48 h and a decline at 72 h (Fig. 4C). We note that the pattern of highest binding intensity at 48 h and a decline in intensity at 72 h was present for most of the cell wall-related mutants (see below) and is referred to as the “cell wall trend.” Overall, this analysis indicated that Cps1, and therefore presumably hyaluronic acid, also influenced the response to carbon limitation at the levels of capsule permeability and cell wall accessibility.

**Fig 5:**
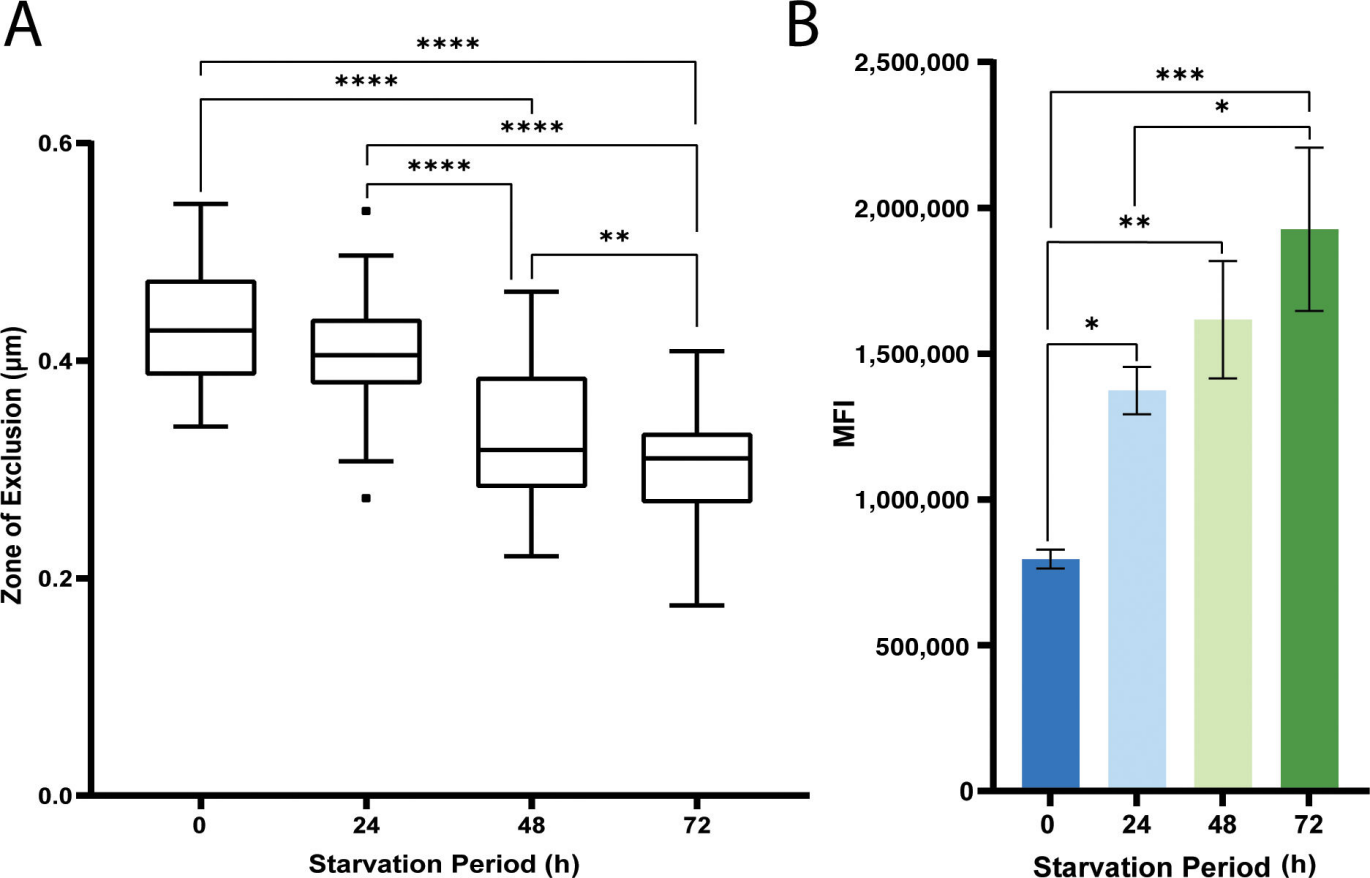
The *pbx2*Δ mutant displays a trend in capsule structure similar to WT upon carbon limitation. The dextran permeation assay is shown on the left and the CFW binding assay is shown on the right. Statistical significance was measured with an ordinary one-way ANOVA, and the labeled significance was based on the results of the post-hoc comparisons if the *P*-value was statistically significant in the ANOVA summary (^*^P<0.05, ^**^P<0.01, ^***^P<0.001, ^****^P<0.0001).

An additional cell wall-related gene, *PBX2,* was also studied using an available *pbx2*Δ mutant. Pbx2 is a parallel β-helix protein that plays a role in surface glycan synthesis and acts in remodeling the cell wall to maintain a normal cell morphology and precursor availability for other glycan synthetic processes (53). *C. neoformans* contains two parallel β-helix proteins with redundant functions; single deletions of the genes encoding the Pbx proteins do not have extreme cell wall defects, aside from melanin retention. However, the mutants display defects in capsule structure, and this defect prevents proper attachment of extracellular polysaccharide isolated from the *pbx2*Δ mutant to an acapsular strain (*cap59Δ)* (53). The capsule of the *pbx2*Δ mutant has a looser structure, but a similar diameter to the WT strain, and this is an important feature relevant to the assays used in our study (53, 54). In particular, the *pbx2*Δ mutant showed similar trends to the WT strain (Fig. 5), revealing that despite the looser structure, the capsule gradually became less dense as the starvation period continued and the porosity increased in the same fashion. A notable difference between the mutant and WT was the intensity of binding in the CFW binding assay with over double the intensity at each measured time point in the mutant; this phenotype may be explained by the looser structure of the capsule at the start of the starvation period.

### Loss of Kre64 blocks changes in capsule permeability upon carbon limitation

**Fig 6:**
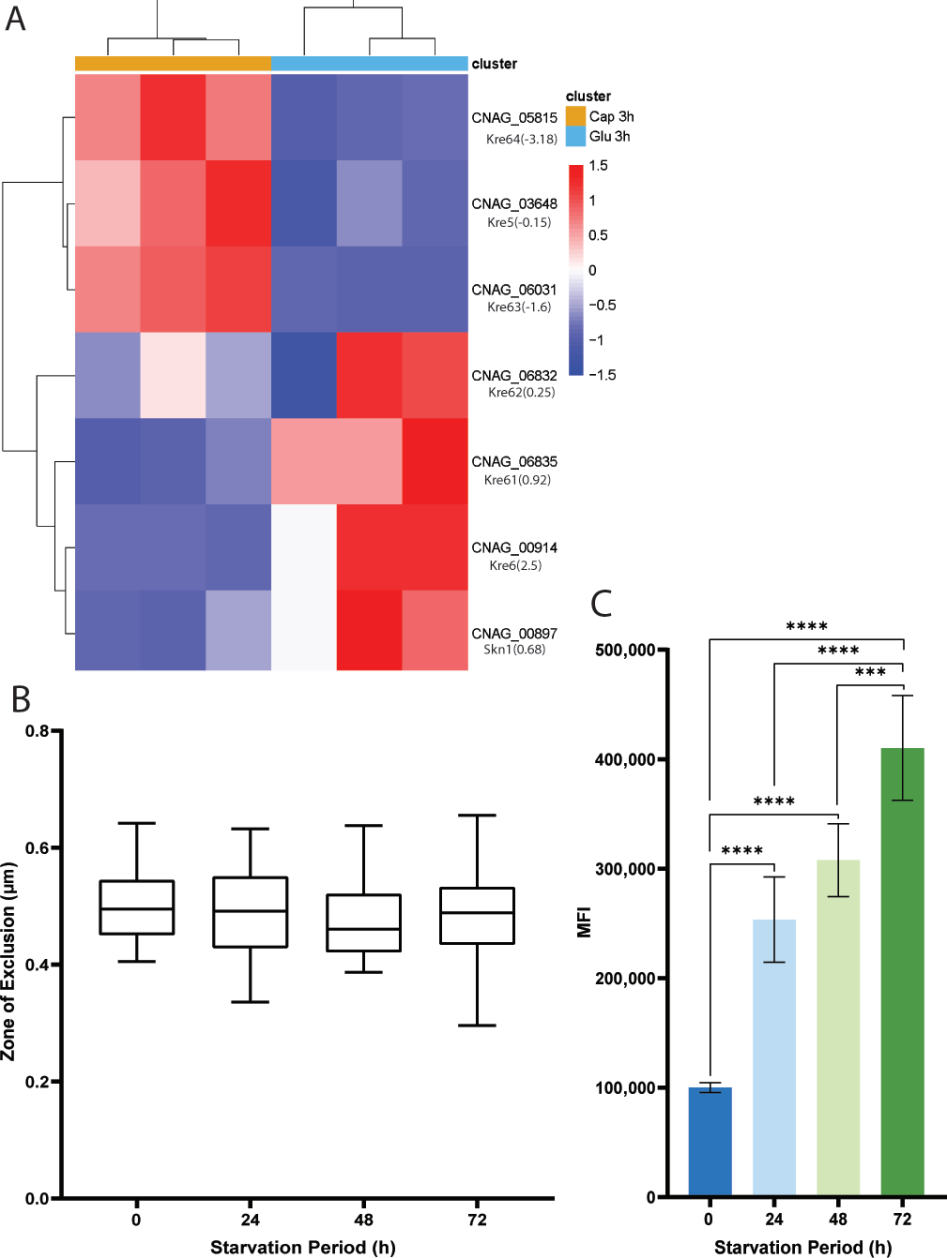
A *kre64*Δ mutant is impervious to dextran permeation of the capsule during carbon starvation. (**A**) Heatmap of the differential expression of genes in the Kre6/Skn1 group with the log2-fold value indicated in parentheses. The three replicates of the starvation condition are labeled orange, and the glucose condition replicates are labeled blue. (**B**) The dextran permeation assay for the *kre64*Δ mutant is shown on the left. (**C**) The results for the CFW binding assay for the mutant are shown on the right. Statistical significance was measured with an ordinary one-way ANOVA, and the labeled significance was based on the results of the post-hoc comparisons if the *P*-value was statistically significant in the ANOVA summary (^*^P<0.05, ^**^P<0.01, ^***^P<0.001, ^****^P<0.0001).

The Kre6/Skn1 pathway is involved in the synthesis of β−1,6-glucan and modification of the polymer in the cell wall (36). The genes in this pathway (encoding Kre5, Kre6, Skn1, Kre61, and Kre62) are functionally redundant with single deletion mutants having phenotypes indistinguishable from the WT (36). However, there are unique features and functions for some of the proteins that may be relevant to capsule and cell wall formation. For example, Kre63 and Kre64 have the major Skn1 domain, but neither has a transmembrane-spanning domain, and Kre64 is the only member of the group with a concanavalin A-like domain (36). The latter domain is a lectin that binds specifically to various sugars, glycoproteins, and glycolipids (55). The transcripts for *KRE63* and *KRE64* were both upregulated in the carbon limitation condition, and *KRE6* was upregulated in the glucose condition (Table 3 and Fig. 6A). We tested the *kre64*Δ mutant in the dextran permeation assay and found no difference between the starved and non-starved samples, indicating an impact on responsiveness (Fig. 6B). The CFW binding assay revealed a high level of staining at all of the time points, presumably reflecting a change in cell wall composition (Fig. 6C). These results support the idea that the Kre64 enzyme (and presumably the Kre63 enzyme) could be involved in the modification process of the β−1,6-glucan present in the cell wall or capsule material during carbon starvation.

**TABLE 3:** Known genes involved in the Kre/Skn1 pathway, inferred from homology with *Saccharomyces cerevisiae,* along with their expression values in the RNA-seq data, a positive value being differential expression favoring glucose reintroduction and a negative value favoring the starvation condition

Gene	log2FoldChange	Function of the gene product
*KRE64*	−3.18	Concanavalin α-like lectins/glucanases 1
*KRE63*	−1.6	β-glucan synthesis-associated protein
*KRE6*	2.5	Involved in β−1,6-glucan synthesis, cis-Golgi type II transmembrane protein
*KRE5*	−0.15	Involved in β−1,6-glucan synthesis, homology to mammalian UDP-glucose glycoprotein glycosyltransferases (UGGTs)
*KRE61*	0.92	Glucosidase
*KRE62*	0.25	Glucosidase
*SKN1*	0.68	Involved in β−1,6-glucan synthesis, *cis*-Golgi type II transmembrane protein

### Specific chitin synthases impact capsule permeability

**Fig 7:**
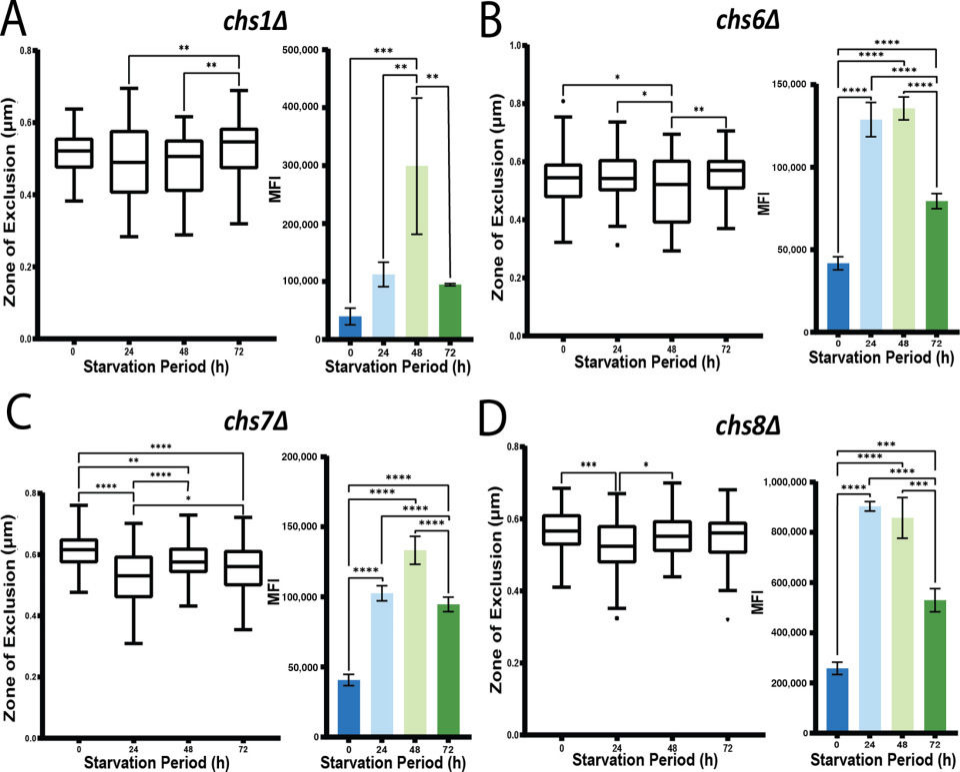
Chitin synthase mutants have altered responses to carbon starvation. (**A-D**): Assays of dextran permeability (left) and CFW binding (right) are shown for each indicated mutant. Note that the dextran permeability assay results were variable between mutants, suggesting different contributions of the chitin synthases. Statistical significance was measured using an ordinary one-way ANOVA, and the labeled significance was based on the results of the post-hoc comparisons if the *P*-value was statistically significant in the ANOVA summary (*P<0.05, **P<0.01, ***P<0.001, ****P<0.0001).

**Fig 8:**
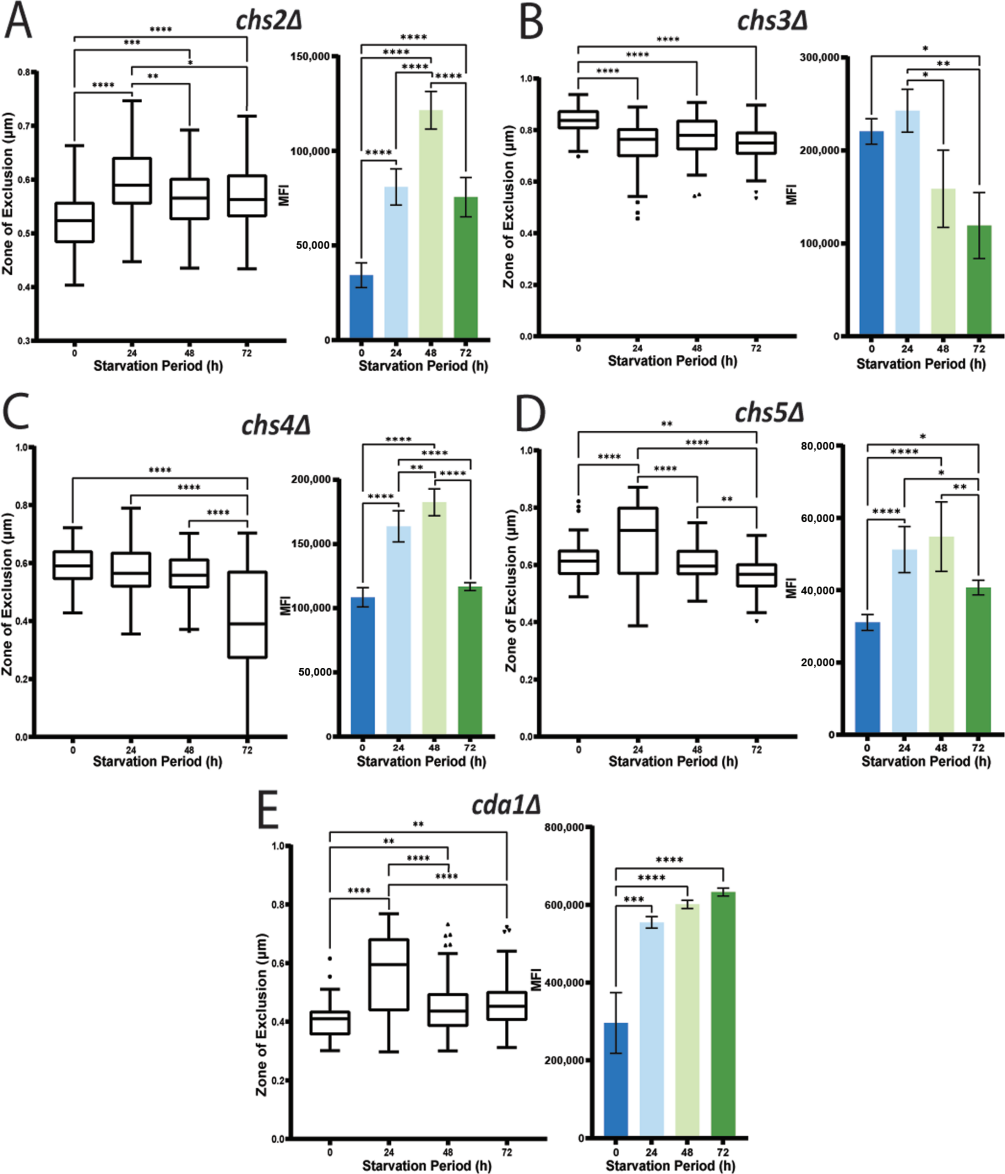
Additional chitin synthase mutants and a chitin deacetylase mutant have altered responses to carbon starvation. (**A-D**) Assays for dextran permeability (left) and CFW binding (right) are shown for each of the mutants. Note that the dextran permeability assay results were variable between mutants, suggesting different contributions of the chitin synthases. (**E**) Analysis of the *cda1*Δ mutant with the dextran permeation assay (left) and the CFW binding assay (right). Statistical significance was measured with an ordinary one-way ANOVA, and the labeled significance was based on the results of the post-hoc comparisons if the *P*-value was statistically significant in the ANOVA summary (*P<0.05, **P<0.01, ***P<0.001, ****P<0.0001).

Chitin is a major component of the cell wall and also contributes to the stability of GXM fibers in their attachment to the capsule (45, 56). Chitin synthases transfer the sugar moiety of UDP-GlcNAc to the non-reducing end of the growing chitin polymer (44). *C. neoformans* contains eight chitin synthases, spanning multiple classes and showing differential expression. Along with the *chs2*Δ mutant, we examined each of the other chitin synthase mutants in our assays to assess their contributions to the response to carbon starvation (Fig. 7A through D , and 8A through D). With the exception of the *chs3*Δ and *chs8*Δ mutants, each of the other mutants showed a similar pattern of CFW staining upon carbon limitation with an initial increase followed by a drop at 72 h. The *chs3*Δ and *chs8*Δ mutants resembled the WT level of staining at 24 h and then showed a decrease at 48 and 72 h (Fig. 7D and 8B). For the permeation assay, the phenotypes of the different mutants fell into three categories. In the first category, represented by the *chs1Δ, 6Δ, 7*Δ, and *8*Δ mutants, the dextran permeation assay revealed only a small or no decrease in the zone of exclusion during the first 24 h of carbon limitation, and no change as starvation continued (Fig. 7A through D). This trend was similar to the absence of a change in permeability observed for the *cps1*Δ, *cas3*Δ, and *kre64*Δ mutants (Table 2). The second category represented by the *chs3*Δ and *chs4*Δ mutants followed the expected trend of decreased zones of exclusion as carbon starvation continued (Fig. 8B and C). The third and most interesting category included *chs2*Δ and *chs5*Δ, which had decreased permeability (Fig. 8A and D), suggesting that the defects in these strains actually reversed the influence of starvation on dextran access. An interesting trend was that the most obvious differences between the mutants and WT were observed primarily at the 24 h starvation point (except for *chs4*Δ), suggesting that this point may be a key time for the influence of the cell wall-related mutations, as also seen with the *cps1*Δ mutant.

We also examined another chitin-related mutant defective in Cda1, one of three chitin deacetylases in *C. neoformans*. These proteins have redundant function, and a triple gene deletion is required to impair chitosan production (57, 58). The *cda1*Δ mutant had a slight increase from non-starved cells to the starved samples with a significant increase in the zone of exclusion at 24 h (Fig. 8E). The CFW binding assay had a similar trend to the WT with a gradual increase in MFI from the non-starved level as carbon limitation continued (Fig. 8E). However, a full understanding of the effect of chitosan on the response to carbon limitation will require an analysis of the triple deletion mutant.

### The cell wall undergoes modification during carbon starvation

**Fig 9:**
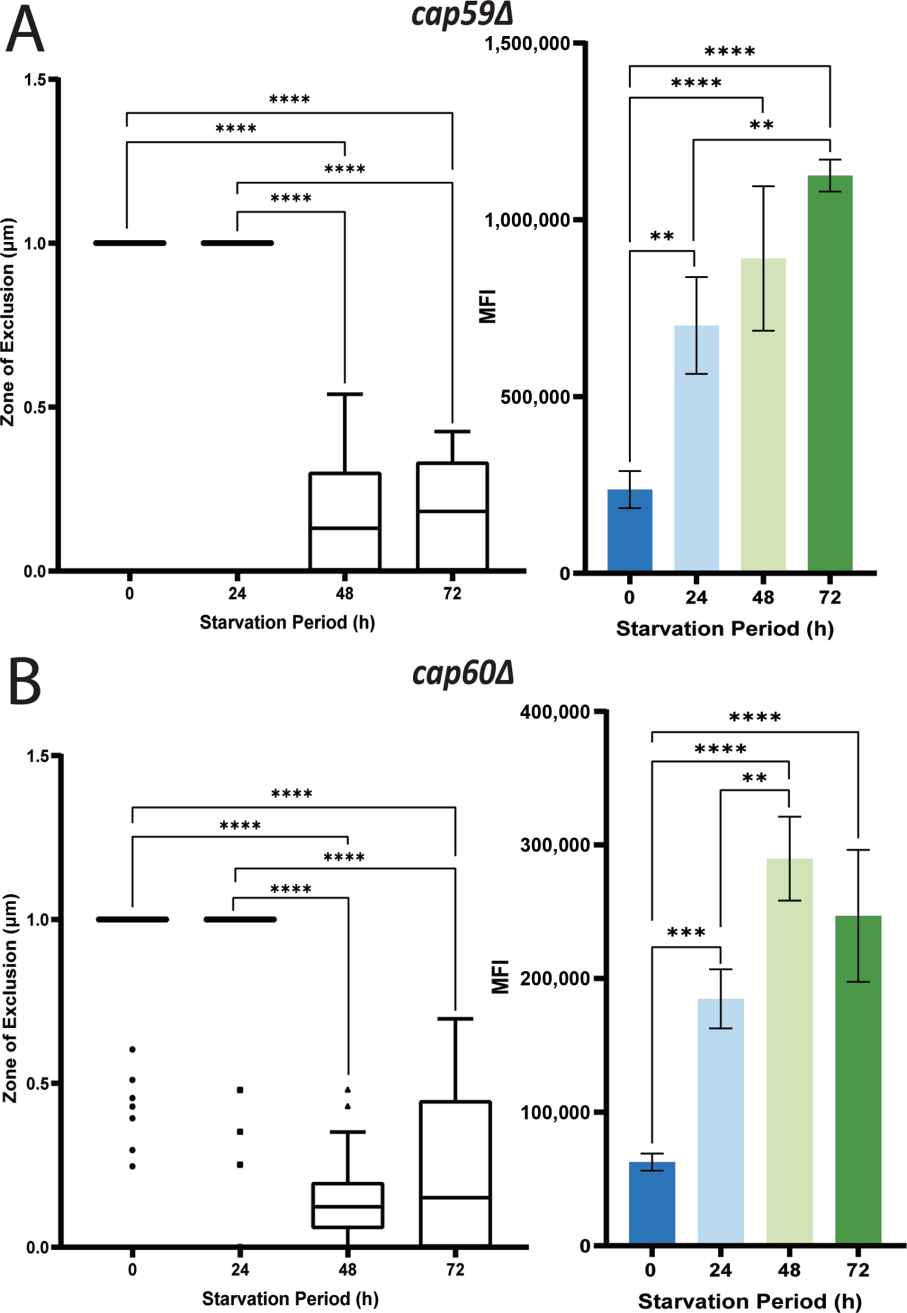
Acapsular mutants show altered cell walls upon carbon limitation. Analysis of the *cap59*Δ mutant (**A**) and the *cap60*Δ mutant (**B**) with the dextran permeability assay (left) and the CFW binding assay (right). Note that the CFW binding assay (right) for the *cap60*Δ mutant showed the same trend as the chitin synthase mutants, and the dextran permeation assay was similar for the *cap60*Δ and *cap59*Δ mutants (i.e., full dextran permeation after 24 h). Statistical significance was measured with an ordinary one-way ANOVA, and the labeled significance was based on the results of the post-hoc comparisons if the *P*-value was statistically significant in the ANOVA summary (^*^P<0.05, ^**^P<0.01, ^***^P<0.001, ^****^P<0.0001).

The cell wall and capsule have an important relationship for supporting virulence such that acapsular mutants are avirulent in a murine model and chitin synthases are known to influence capsule (45). We employed two acapsular mutants, *cap59*Δ and *cap60*Δ, in the assays to specifically observe the response to dextran permeability and CFW binding upon carbon starvation (Fig. 9). Cap59 is an α−1,3-mannosyltransferase and part of the GT69 family. This protein is responsible for the mannosylation of the backbone of GXM. Cap59, Cmt1, and Cap6 all contain a DXD motif, with a glycine residue at the center important for enzymatic function (6). The *cap59*Δ mutant does not produce a capsule but still produces and secretes exopolysaccharide, and the mutant is completely avirulent in a murine model (59). The dextran assay showed a full cell zone of exclusion for the non-starved cells and for 24 h starved cells. However, the 48 h and 72 h starved cells had almost complete permeation by the dextran with the zone of exclusion being 0.1704 μm and 0.1792 μm, respectively (Fig. 9A). The CFW binding assay was similar to the WT with an increase in MFI at each of the starvation points; however, the intensity of binding was generally lower in the WT. These data suggest that the cell wall is undergoing a compromising alteration during carbon starvation after 24 h, allowing for the dextran to permeate the cell wall entirely, since there is no capsule present.

Cap60 shares homology with Cap59 but lacks a signal peptide, and the mutant is also acapsular and avirulent. Proteins encoded by Cap60 and Cap59 have 45% similarity and 22% identity with most conserved regions in the central portions (6). *CAP60* and *CEL1* are also closely linked with *CEL1* being immediately upstream and transcribed in the same direction (6). Unlike *CAP59*, the *CAP60* transcript had a 2.21-fold change in the glucose condition. The dextran assay for the *cap60*Δ mutant was similar to that of the *cap59*Δ mutant with full zones of exclusion at the non-starved and 24 h times, and a significant decrease in the zone of exclusion at 48 and 72 h (Fig. 9B). As shown in Fig. S9 for both mutants, images of the cells revealed little to no permeation for the non-starved cells compared with nearly complete permeation for the starved cells by 72 h. The CFW binding assay followed the “cell wall trend” with a high level at 48 h and a reduced MFI intensity compared with the *cap59*Δ mutant (Fig. 9B). The only other significant result was that an increase in MFI occurred as starvation continued between 24 and 48 h. The analysis of these mutants suggested that the cell wall is also undergoing a compromising modification around 48 h, which allowed full permeation of the TMR dextran into the interior of the cell, a phenotype not seen with the cells containing a capsule. The CFW binding intensity results are consistent with alterations in the cell wall during starvation.

## DISCUSSION

Producing the capsule is a resource-intensive process for *C. neoformans*, and the fungus must allocate substantial carbon resources to construct the large polysaccharide structure. It is unknown whether the fungus is able to reuse sugars from the capsule or whether there are processes in place to modify the existing capsular components in response to different carbon sources or limitation. Here, we used TMR dextran permeation measurements to discover that glucose starvation was associated with a reduction in capsule density. Furthermore, we found that CFW binding increased as starvation progressed, suggesting modifications to wall composition and secondarily a change in capsule structure leading to greater cell wall access. Imaging by TEM indicated that the cell wall and the cell wall-capsule interface are modified during carbon starvation, seen particularly after 48 and 72 h. SEM images of cells also supported the conclusion that the porosity of the capsule changed during carbon starvation. An interesting feature of these images was the visible reduction of branching present at the 72 h time point. This result supports the conclusions from the permeability assays and also suggests that the mechanism involved in the capsule alterations could target branching components of the structure instead of the backbone.

Starvation conditions were associated with greater capsule permeability as indicated by increased penetration of both dextran and India ink particles. Although the architecture of the capsule is not fully understood, analysis of capsular polysaccharides suggests that these are large, branched macromolecules, which presumably form a lattice that can allow small molecule nutrients and waste products to diffuse freely. Although DLS analysis of sonication-shed polysaccharide showed that the major components were unchanged, we detected the appearance of minor populations of smaller and larger particles in starved cells at time points associated with increased permeability. The changes in particle populations and sizes indicate that the capsule is a dynamic structure that is remodeled in conditions of nutritional stress. It may be that only relatively small changes to the polysaccharide lattice are actually needed to increase its permeability to large particles. The emergence of a minor polysaccharide population during starvation raises the possibility that some of the capsule components could be rearranged to be metabolized for nutrition, although confirmation of this idea would require metabolic studies. The nutritional and physical properties of the host environment may also impact capsule structure as demonstrated by Geunes-Boyer et al. (60). This study revealed that cryptococcal cells isolated from the lungs of mice lacking the surfactant protein D displayed a “leaky” or less dense capsule morphology similar to the phenotype we observed for starved cells (Fig. 1A). Together, these results suggest that host conditions that modify capsular architecture could impact disease progression, for example, by influencing uptake by alveolar macrophages.

We coupled our capsule and cell wall assays with an RNA-seq experiment to specifically compare the transcriptome differences between carbon starvation and glucose reintroduction. The observed differential transcripts identified a general response of the Ras signaling pathway, as well as more specific regulation of cell wall and capsule functions (i.e., Chs2, Cas3, Cap60, and Kre64). These results are consistent with a study by Maeng et al. in which a transcriptomic analysis revealed that the Ras signaling pathway (Ras1) maintained cell wall integrity in a Cdc24-dependent manner (46). This study also found that the *ras1*Δ mutant exhibited an increased susceptibility to hyperosmotic stress imposed by high KCl and NaCl in both rich and poor glucose conditions, which was not found with other mutants like *aca1*Δ, *cac1*Δ, or *pka1*Δ (46). The increased expression of Ras signaling pathway components in our study further supports a role for the pathway in remodeling upon carbon limitation, specifically with regard to the cell wall or chitin, as seen with the increase in Chs2, Chs6, and Cps1 transcripts. In general, our RNA-seq analysis revealed starvation-related responses that prompted the hypothesis that modifications in cell wall components could mechanistically drive the measured changes to capsule permeability and density. An increase in the transcripts of several functions for glucan and chitin biosynthesis was observed with the reintroduction of glucose, suggesting that remodeling is needed post-starvation; interestingly, these functions included the Cap60 protein involved in capsule production. Subsequent assays of mutants for the capsule-related genes also suggested a relationship between the capsule and cell wall that is important for a proposed degradative process that influences capsule integrity.

The influence of carbon source on *C. neoformans* morphogenesis was studied previously using transcriptome analysis (61, 62). For example, Denham et al. compared the transcriptome of cells in YNB (yeast nitrogen base medium), capsule-inducing medium (CIM, low glucose), and cells grown in CIM and then exposed to media containing unspent nutrients and secreted fungal factors (61). That study found that phosphate ion transport genes were induced in cells grown in CIM but suppressed in both YNB and conditioned media. These results indicated that phosphate is a critical signal for the small cell induction that is present when cells are grown in CIM. Their analysis also revealed through GO term enrichment analysis that the top enriched GO term upregulated in the CIM condition was oxidation-reduction processes, which was also the top condition in our analysis of the starvation condition (61). Another study found differential gene expression between a variety of conditions including YPD, capsule-inducing media, pigeon guano, and within macrophages, and this analysis led to the discovery of five notable gene clusters (62). One cluster contained genes upregulated in YPD and CIM that were mainly involved in vesicle-mediated transport, carbohydrate metabolism, and lipid metabolism; this cluster was primarily associated with cell wall and/or capsule production. In their *in vivo* condition, a cluster was identified with ~120 virulence-associated genes, and half of them were involved in capsule formation and chitin synthesis and regulation (62). The results from these studies indicate changes in the expression of genes encoding capsule and cell wall-specific proteins in capsule-inducing media (CIM) and a rich medium (YPD), as well as in response to high and low glucose (63). We note that the CIM used in these studies contained a lower concentration of glucose than a nutrient-rich media, and the resulting patterns only partially reflect the response to glucose, in contrast to our focus on complete carbon limitation.

Due to the intrinsic relationship between cell wall and capsule, the functions that we tested for a response to carbon limitation may have complex and/or indirect influences on capsule structure. The results from mutants with defects in chitin synthase or chitin deacetylase suggest that these enzymes are not entirely functionally redundant. This idea is also supported by the results from the *cas3*Δ mutant which has (i) an altered capsule structure, (ii) greater abundance of an additional moiety that included an extra xylose residue, (iii) partly 6-O-acetylated mannose residues, and (iv) half the acetylation ratio of the parent strain (48, 49). It appears that acetylation may play a role in the response to carbon starvation because we found that *CAS3* and *CAS1,* which is also involved in O-acetylation of the capsule (48), were both upregulated upon glucose reintroduction, although the *cas1*Δ mutant behaved more like the WT strain in our permeability assays.

The observed regulation of *CHS2* in our transcriptome data prompted an evaluation of all of the mutants for chitin synthase genes for a potential impact on the response to stress and carbon limitation (32, 44, 45). Some of the mutants had delayed responses to starvation compared with the WT, such as with *chs4*Δ, which only displayed an increase in permeation at 72 h of starvation. These structural differences could lead to minimal access to the capsule or capsular component that the enzymes involved in this process require for modification or that the carbon limitation then triggers expression of enzymes that influence remodeling differently in the mutant than in the WT. Many of the chitin synthase mutants displayed a peculiar trend in CFW binding, which initially increased upon carbon limitation and then decreased at 72 h. This result may suggest alternative function(s) for these enzymes or, perhaps, that they act in conjunction with other cell components acting later in the starvation response. Published data support this finding since each of the chitin synthases displays an array of phenotypic differences when lost. For example, there are differences in extracellular vesicle (EV) trafficking/production, and EVs are overproduced in *chs4*Δ and *chs5*Δ mutants and underproduced in *chs2*Δ, *chs3*Δ, and *chs6*Δ mutants (45). The mutants also have variability in capsule structure, including fiber density differences (*chs1Δ, chs3Δ, chs4Δ, chs5Δ, chs6Δ,* and *chs8*Δ mutants) and polarized sections of the cell creating clumps of attached capsular polysaccharide (*chs2Δ, chs4Δ, chs5Δ*, and *chs6*Δ mutants) (45). In our assays, the *chs3*Δ and *chs8*Δ mutants differed from the other chitin synthase mutants, providing evidence for additional functions outside of synthesizing chitin.

The *chs2*Δ and *chs3*Δ mutants illustrate the contributions of specific chitin synthases in the response to carbon limitation. The *chs2*Δ mutant lacking a class three chitin synthase leads to lower levels of extracellular vesicles and altered intracellular chitinase activity (45). As noted, this mutant followed the CFW binding trend seen in most of the chitin synthase mutants. The zone of exclusion for the dextran permeability indicated that Chs2 might play a larger role in modifications to the capsule or cell wall since the zone of exclusion increased as the starvation continued. Compared with other *chs*Δ mutants, the *chs2*Δ mutant has a larger polysaccharide diameter and a denser capsule but still contains a polarized distribution of polysaccharide fibers (45), which could explain the difference between this chitin synthase and the other chitin synthase mutants. The *chs2*Δ mutant also demonstrated an altered chitinase response in stress conditions (45), which, when compared to the *cda1*Δ mutant, showed similar results in the dextran permeation assay, potentially implicating chitosan in this process.

The *chs3*Δ mutant has a reported defect in the α−1,3-glucan required for capsular attachment (44). Chs3 is the most important chitin synthase for vegetative growth and has specific regulation outside of the Ras pathway by Skt5. The *chs3*Δ mutant is the only deletion of a chitin synthase gene that affects the amount of chitin deacetylated to chitosan for vegetatively growing cells. The mutant also has a smaller capsule, smaller polysaccharide fibers, and underproduces extracellular vesicles (45). Our CFW binding assay suggested that the dynamic between the cell wall and capsule plays a role in the carbon starvation response, through α-glucan attachment, general cell wall modifications, or as seen with the *cps1*Δ mutant (with a defect in hyaluronic acid synthase), other non-GXM or GalXM components could be vital in this process.

The results for the dextran permeation assay with the *cap59*Δ and *cap60*Δ mutants illustrated an additional response to starvation, specifically outside of capsule formation, with the dextran permeating most, if not all, of the cell. This was not observed with the non-starved or 24 h samples and suggests a cell wall-specific response to carbon starvation that is delayed vs. that of the capsule-specific response. For example, a starvation response may work in parallel with the HOG stress response pathway to maintain osmotic balance within the cell, which was most likely hindered by the changes at the cell wall in the acapsular mutants.

Our findings are consistent with the impact of carbon source on the cell wall identified in other fungi. For example, Ballou et al. discovered that *C. albicans* and other *Candida* species modulate the exposure of β-glucan at the cell surface in response to carbon sources (e.g., lactate versus glucose) and in a manner depending on a lactate sensor Gpr1 and the Crz1 transcription factor (64). This lactate-influenced exposure is important for interactions with the host immune system, given the role of β-glucans as pathogen-associated molecular patterns (PAMPs) that trigger dectin-l mediated recognition (64). In this regard, it would be interesting to know if carbon limitation results in exposure of polysaccharide or other PAMPs within the capsule of *C. neoformans*. It is known that mannoproteins in the capsule are immunogenic, and the abundance of these proteins may be altered during carbon starvation (65). For example, expression of the Cig1 mannoprotein is induced by iron starvation and is involved in virulence and heme uptake (65). Additionally, a lactonohydrolase (Lhc1) is an interesting candidate for a responsive capsule-associated protein that is potentially responsive to nutritional changes (66). This idea comes from the observation that a mutant lacking Lhc1 is altered in capsule branching and density, and a proteomics study of the secretome indicating that Lhc1 is abundant upon induction of protein kinase A expression (66, 67). The transcript for *LHC1* was upregulated (+2.38) in the RNA-seq data, following the expected results for expression being regulated based on nutritional changes from Park et al. (66). Additional support comes from our analysis of a mutant lacking Cap6, which is involved in protein O-mannosylation and regulated by the MPK1-CWI pathway (68). Cap6 plays a role in cell morphology and the secretion and degradation of proteins. The *cap6D* mutant had a slight increase in the zone of exclusion in the dextran permeability assay, perhaps because of redundance with other O-mannosylation functions; however, the regulation of the *CAP6* gene was less than 1 log2-fold in the RNA-seq data. Further identification of secreted proteins that appear during starvation may also be informative. The CAZymes identified in the RNA-seq top 100 genes are ideal candidates to functionally test for their potential effects on the capsule and cell wall due to their high expression during starvation. Determining the enzymes responsible could also potentially be used for capsule degradation outside of starvation conditions as a method for compromising specific components of the capsule or cell wall to decrease the virulence of *C. neoformans*.

In summary, our study revealed increases in dextran permeability of the capsule and CFW binding to the cell wall in response to carbon limitation. Increased permeability could give the fungal cell access to additional sources of nutrition, and this phenomenon is associated with changes to the capsular polysaccharide. The use of specific mutants defective in biosynthetic functions reinforced the close association between the cell wall and capsule structure. It would be informative to perform the assays upon carbon starvation with a broader range of mutants to develop a more comprehensive view of the functions that mediate capsule structure. Additionally, our RNA-seq experiment only used samples that were starved for 24 h, and based on the results, a more extreme carbon starvation response was found after 48 h. Therefore, a longer starvation period along with the use of different starvation conditions (e.g., carbon and nitrogen limitation) could reveal additional potential mechanisms of adaptation to starvation.

## MATERIALS AND METHODS

### Strains, media, and primers

Strains used for all experiments were derivatives of *C. neoformans* var. *grubii* (serotype A) strain H99 (H99S and KN99α). Other strains are listed in Table S3. Strains were propagated and maintained on a YPD-rich medium (1% yeast extract, 2% peptone, 2% dextrose, and 2% agar). Overnight cultures were grown in liquid YPD medium in a shaking incubator at 30°C and 180 rpm. Iron-chelated dH_2_O was prepared by passage of dH_2_O through a column containing Chelex-100 resin (BIORAD Chelex-100) and used in the preparation of capsule inducing media (CIM) (0.5% glucose, 38 mM L-asparagine, 2.3 mM K_2_HPO4, 1.7 mM CaCl_2_·2H_2_O, 0.3 mM MgSO_4_·7H_2_O, 20 mM HEPES, and 22 mM NaHCO_3_), 1 ml of 1,000× salt solution (0.005 g/L CuSO_4_·5H_2_O, 2 g/L ZnSO_4_·7H_2_O, 0.01 g/L MnCl_2_·4H_2_O, 0.46 g/L sodium molybdate, and 0.057 g/L boric acid), in iron-chelated dH_2_O adjusted to pH 7.4 with 0.4 mg/L sterile thiamine added post-filtering. Starvation-inducing media contained the same components as CIM without the 0.5% glucose.

The mutants for screening, listed in Table S3, were generated in previous studies in the Kronstad laboratory through biolistic transformation or obtained from the *C. neoformans* nonessential gene knockout strain collection generated in the Madhani Laboratory (69). The collection was obtained from the Fungal Genetics Stock Center (https://www.fgsc.net/), and the mutants were independently confirmed by PCR. All of the deletion mutants are nourseothricin (NAT) resistant and were propagated on YPD; the parental strain KN99α is derived from the H99 clinical isolate of *C. neoformans* var. *grubii* (serotype A) and is a prototroph.

### RNA-seq analysis

Cells for RNA-seq sample preparation were starved for 24 h in no-carbon media with or without the reintroduction of glucose into the starvation media for 3 h. RNA was then isolated with the RNeasy kit for RNA preparation kit (QIAGEN). Total RNA was sent for cDNA library preparation and sequencing at the Biomedical Research Center at the University of British Columbia, and the libraries were sequenced on the HiSeq platform with 1 × 50 bp configuration (South Plainfield, NJ, USA). BBDuk (http://jgi.doe.gov/data-and-tools/bb-tools) was used to filter paired reads for Illumina adapter content prior to alignment to the *C. neoformans* (Accession: PRJNA411) genome using the STAR genome aligner (--quantMode GeneCounts) (70). Gene counts from STAR were then processed in R for differential gene analysis. Determination of differentially expressed genes was performed using the DESeq2_1.34.0 package (71) and visualized in volcano plots, heatmaps, and by principal component analysis.

### RNA-seq pathway analysis

To identify significantly overrepresented functional groups within the up- and down-regulated genes, H99 gene identifiers (IDs) were first converted to gene IDs from the genome of *C. neoformans* strain JEC21 using FungiDB to establish compatibility to KEGG database annotations (72). Gene set enrichment analysis (GSEA) was then used to identify enriched pathways based on a ranked gene list generated from the DESeq2 output and pathway information from the KEGG database (73). GSEA was carried out at 10,000 permutations and included gene sets between 10 and 400. An enrichment map was then used to visualize the results at a Benjamini-Hochberg FDR value of 0.25 and *P*-value cutoff of 0.05 for all comparisons (74). Graphs were created using GO enrichment analysis (75, 76) (https://geneontology.org/docs/go-enrichment-analysis/) and used all the genes with a log2 fold change of one and above and a *P* value<0.05. The RNA-Seq data were deposited in the European Nucleotide Archive (ENA) (PRJEB98634).

### Preparation of cells for identifying capsule and cell wall changes

Cells were grown in liquid YPD medium in a shaking incubator at 30°C and 180 rpm overnight. The cells were then washed twice with low-iron H_2_O and moved to CIM in a shaking incubator at 30°C for 48 h. The cells were then washed with low-iron H_2_O and moved to no-carbon CIM for the allotted starvation time at 30°C. Start times for the cells were staggered so that all starvation times (24, 48, and 72 h) would end at the same time to allow experiments to be conducted simultaneously. Cells were washed and fixed with 3.7% paraformaldehyde (PFA) and incubated at room temperature for 30 min with mixing by inverting every 5 min.

### Calcofluor white flow cytometry

Fixed cells were washed twice with phosphate-buffered saline (PBS) and adjusted to 10^6^ cell/mL. Cells were incubated in 0.0001 μg/mL of calcofluor white (CFW) in PBS for 5 min at 25°C. Cells were washed twice with PBS and processed by flow cytometry. Fluorescence was quantitated on a Cytek Aurora flow cytometer (Cytek Biosciences), and the data were analyzed using FlowJo v. 10.6.2. For the analysis, cells were first gated for single cells to avoid budding cells and clumps of cells; single cells were then gated for FSC × SSC, followed by the analysis of FSC × CFW. The intensity of CFW was assessed by mean fluorescent intensity (MFI).

### Flow cytometry with mAb 18B7

Cells grown in the previously described conditions were washed once with PBS and counted. Two million cells were then incubated with the anti-capsule monoclonal antibody mAb 18B7. Cells were washed again three times with PBS and incubated with the secondary antibody, anti-mouse 488 Alexa fluor, for 1 h. Cells were again washed three times in PBS, resuspended, and analyzed on a Cytoflex L Analyzer, and the data were analyzed using FlowJo v. 10.6.2. The binding of mAB 19b7 was imaged on a Leica STELLARIS 5 confocal microscope equipped with laser lines for 488 nm and a HC PL APO 63×/1.40 OIL CS2 objective. The LAS X Lightning Expert software was used for additional image deconvolution.

### Dextran permeability assay

Tetramethylrhodamine (TMR)-labeled dextrans (Molecular Probes) with average molecular masses of 10 kDa (D-1816), 40 kDa (D-1842), 70 kDa (D-1819), and 2,000 kDa (D-7139) were used to assess the permeability of the capsule to macromolecules with various Stokes radii. The estimates of Stokes radii for the dextrans were from a published report (13, 28). *C. neoformans* cells (4 × 10^5^ mL^−1^) were incubated with the dextran solutions (1 mg mL^−1^ PBS) in a 50 µL reaction volume for 60 min at room temperature in the dark. The suspensions of yeast cells in the dextran solutions were then examined immediately by fluorescence microscopy using an Axio Imager 2 microscope (Zeiss) with a 100× oil immersion objective. Digital images were captured with an ORCA-flash4.0 LT camera (Hamamatsu), and all image analysis was done with ImageJ. A total of 20 cells per time point were imaged to measure the zone of exclusion. This zone of exclusion was measured by subtracting the radius of the dark inner ring from the visible outer ring around the cell, and this value was then normalized by creating a ratio of the zone of exclusion to cell size, with one being no permeation of the dextran and 0 being fully permeated (measurements are illustrated in Fig. S1).

### Serial dilution spot assays

Cells from overnight cultures were washed twice in PBS, and cell numbers were adjusted to 2 × 10^7^ cells mL^−1^. Next, 10-fold serial dilutions were prepared, and 5 μL (covering a range of 10^5^–10^10^ cells) was spotted onto YPD agar supplemented with Congo Red (0.5 mg/mL) or Calcofluor white (1.5 mg/mL). Plates were then incubated at 30°C for 2 days before being photographed, as described previously (77).

### Dynamic light Scattering

*C. neoformans* strain H99 was grown overnight in YPD to stationary phase at 30˚C. Cultures were washed twice in PBS and diluted 1:50 in CIM with 0.5% glucose and incubated for 2 days at 30˚C. For day 0 samples, cells from the CIM culture were washed twice in PBS and resuspended to 1 × 10^7^ cells/mL in 2 mL of PBS. For the starvation experiments, half of the remaining cells were washed twice in PBS and resuspended in the starvation media (CIM without glucose). Aliquots of the culture were taken and processed from both the starvation media culture and the remaining CIM culture on days 1, 2, 3, and 7 and processed in the same manner as the day 0 sample. To remove the capsular polysaccharide, samples were ultrasonicated, as previously described (15, 16). Sonication was performed with a Fisher Scientific Sonic Dismembrator F550 W/ultrasonic Converter (Waltham, Massachusetts) on ice for 30 s at 17 W (power setting 7). This power setting does not lyse the cells but removes the capsule. Samples were then centrifuged at 2,800 × *g* for 4 min to remove cells, and 100 µL of the supernatant was analyzed using Dynamic Light Scattering using a Zeta Potential Analyzer instrument (Brookhaven Instruments) with each replicate being scanned an average of 10 1-min runs. Replicates were averaged together by sorting the combined particle size values from smallest to largest and calculating a 5-row moving average of the particle size values and the respective relative size occurrence values.

### Scanning electron microscopy

Cells were fixed with 0.2% glutaraldehyde and processed on 0.2-μm filters using 0.2M sodium cacodylate buffer; polysaccharides were stabilized using 0.1% ruthenium red and then treated with osmium solution. Cells were dried using Tousimis Autosamdri 815B Critical Point Dryer. Sputter coating was performed with a Leica EM ACE600 Coater using 3.18 nm Au/Pd. Images were taken with a Zeiss Crossbeam350 FIB-SEM with an Oxford XMAX 170 mm EDX detector and Schottky field emission source at a voltage between 2 and 5 kV and working distance of 5.6 mm. Image sorting was done with Blinder (78) to minimize bias and sort images based on fiber density.

### Transmission electron microscopy

Cells were washed three times in PBS and fixed in 2.5% glutaraldehyde in 0.1 M sodium cacodylate pH 7.2. Additional fixation through high-pressure freezing was done using Leica HPM100 High Pressure Freezer. The cells were washed three times with ddH_2_O and dehydrated through sequential washes with a graded concentration series of ethanol into 100% ethanol. After dehydration, the cells were embedded in Spurr’s resin, and 70 nm sections were cut using a Leica Ultramicrotome UCT. Sections were stained with 2% uranyl acetate for 20 min and then by 2% lead citrate for 10 min. Images were taken on a Hitachi 7,600 transmission electron microscope operating at 80 kV, and images were acquired with an AMT XR51 camera. Each cell that was imaged was measured using ImageJ for the cell wall, dense capsule, and full capsule. The measurements of features from the micrographs are illustrated in Fig. S1.

### Long-term incubation of *C. neoformans*

*C. neoformans* strain H99 was inoculated from frozen stocks onto YPD agar plates and incubated at 30°C for 2 days. Colonies were then picked and inoculated into 1 mL YPD or 3 mL minimal medium (MM: 15 mM dextrose, 10 mM MgSO_4_, 29.3 mM KH_2_PO_4_, 13 mM glycine, and 3 µM thymine-HCl at pH 5.5) broth, and incubated overnight at 30 C° with 180 rpm rotation. Every week, the cultures were maintained in the original media or refreshed with 3 mL fresh MM. India Ink images were acquired by mounting 8 µL of each sample with 2 µL India Ink on a glass slide with a coverslip. Images were acquired on an Olympus AX70 microscope with a 40× objective.

### Analysis of cell viability upon carbon starvation

The starved cells from each time point and the non-starved cells grown for 48 h were washed three times with PBS to remove media and any shed material. The cells were normalized to an OD_600_ of 1 after washing. Cells were then incubated in 5 µg/mL of propidium iodide at 37°C in the dark for 30 min. After incubation, cells were again washed twice and resuspended in PBS before analysis with flow cytometry; 50,000 cells per sample were measured with three biological replicates using a Cytoflex L Analyzer, and the data were analyzed using FlowJo v. 10.6.2.

### Glucose reintroduction

Cells from each time point were washed three times with PBS to remove media and any shed capsule material. The OD_600_ of each sample was measured, and cells representing an OD of 0.2 were used to inoculate wells of a 96-well plate with a final volume of 250µL per well. YPD or CIM media were used to test subsequent growth as measured by OD_600_ readings taken every 12 min with shaking at 30°C for 40 h. Cells from each starvation time point (or from the no starvation comparison) were tested in biological triplicates, and each sample had a technical replicate.

### Macrophage uptake assay

Cells starved for 0, 24, 48, or 72 h were washed twice with PBS before opsonization with 10 μg/mL of the 18b7 capsular monoclonal antibody for 1 h at 30°C. A CFW stain was also included to facilitate visualization of the cell wall. In parallel, the J774.A1 murine macrophage-like cell line was seeded in eight chambered slides (100,000 cells/well) and activated with 150 μg/mL PMA for 1 h at 37°C and 5% CO_2_. Following activation, macrophages were infected with yeast at an MOI of 1:10 (macrophage:yeast) for 2 h at 37°C and 5% CO_2_. Infected macrophages were fixed with 2% paraformaldehyde for 15 min, followed by an F-actin phalloidin stain for 30 min. A counterstain was applied to identify extracellular yeast using a secondary antibody (Alexafluor 555) against the 18b7 monoclonal antibody present on the cryptococcal capsule postopsonization. Images were taken with a Leica THUNDER 3D fluorescent microscope equipped with LED illumination and excitation filters for 405, 488, and 555 nm and a HC PL APO 63×/1.40 OIL CS2 objective. The LAS X THUNDER Expert software was used for additional image deconvolution. Images were then scored from three independent replicates to measure at least 350 macrophages per sample and quantified for macrophages containing intracellular yeast cells. The percentage of macrophages containing yeast cells was then determined for each starvation time point. Additional representative images were taken on a Leica STELLARIS 5 confocal microscope equipped with laser lines for 405, 488, and 555 nm and a HC PL APO 63×/1.40 OIL CS2 objective.

### Statistical analyses

Statistical analyses were conducted using GraphPad Prism version 8.4.3, utilizing ordinary one-way ANOVA. Bioassay results were confirmed through biological triplicates and three independent experiments. The results are presented as mean values with standard deviations, with *P*-values indicating significance from Tukey’s multiple comparisons test. Graphs were generated using the statistical software GraphPad Prism, figures were made using Adobe Illustrator, and representative images were chosen to convey key findings visually using ImageJ. All cell measurements were done in ImageJ and statistics were calculated using GraphPad Prism, with a minimum of 20 cells measured per time point for each strain and each experiment. Image sorting for the scanning electron microscopy images was done through a blinder software (specifics in SEM method section).

## SUPPLEMENTAL MATERIAL

Fig. S1 mbio.03701-25-s0001.pdf Measurements of capsule size, permeability, and antibody staining.

Fig. S2 mbio.03701-25-s0002.pdf The wild-type strain H99 is sensitive to cell wall stress upon starvation.

Fig. S3 mbio.03701-25-s0003.pdf Cell viability is not negatively impacted by carbon limitation.

Fig. S4 mbio.03701-25-s0004.pdf Starvation time impacts the rate of recovery of cells when reintroduced into a medium with glucose.

Fig. S5 mbio.03701-25-s0005.pdf A higher percentage of starved cells are phagocytosed by a macrophage-like cell line compared to non-starved cells.

Fig. S6 mbio.03701-25-s0006.pdf Heatmap of the RNA-seq data demonstrating differential expression of specific genes in the glucose and starvation conditions.

Fig. S7 mbio.03701-25-s0007.pdf Heatmap of the RNA-seq data demonstrating differential expression of specific genes in the glucose and starvation conditions.

Fig. S8 mbio.03701-25-s0008.pdf Summary of the RNA-seq data illustrating the difference in gene expression between the glucose and starvation conditions.

Fig. S9 mbio.03701-25-s0009.pdf Representative images of the zone of exclusion for dextran permeability with the acapsular mutants.

Supplemental Tables mbio.03701-25-s0010.pdf Tables S1 to S3.

ASM does not own the copyrights to Supplemental Material that may be linked to, or accessed through, an article. The authors have granted ASM a non-exclusive, world-wide license to publish the Supplemental Material files. Please contact the corresponding author directly for reuse.
