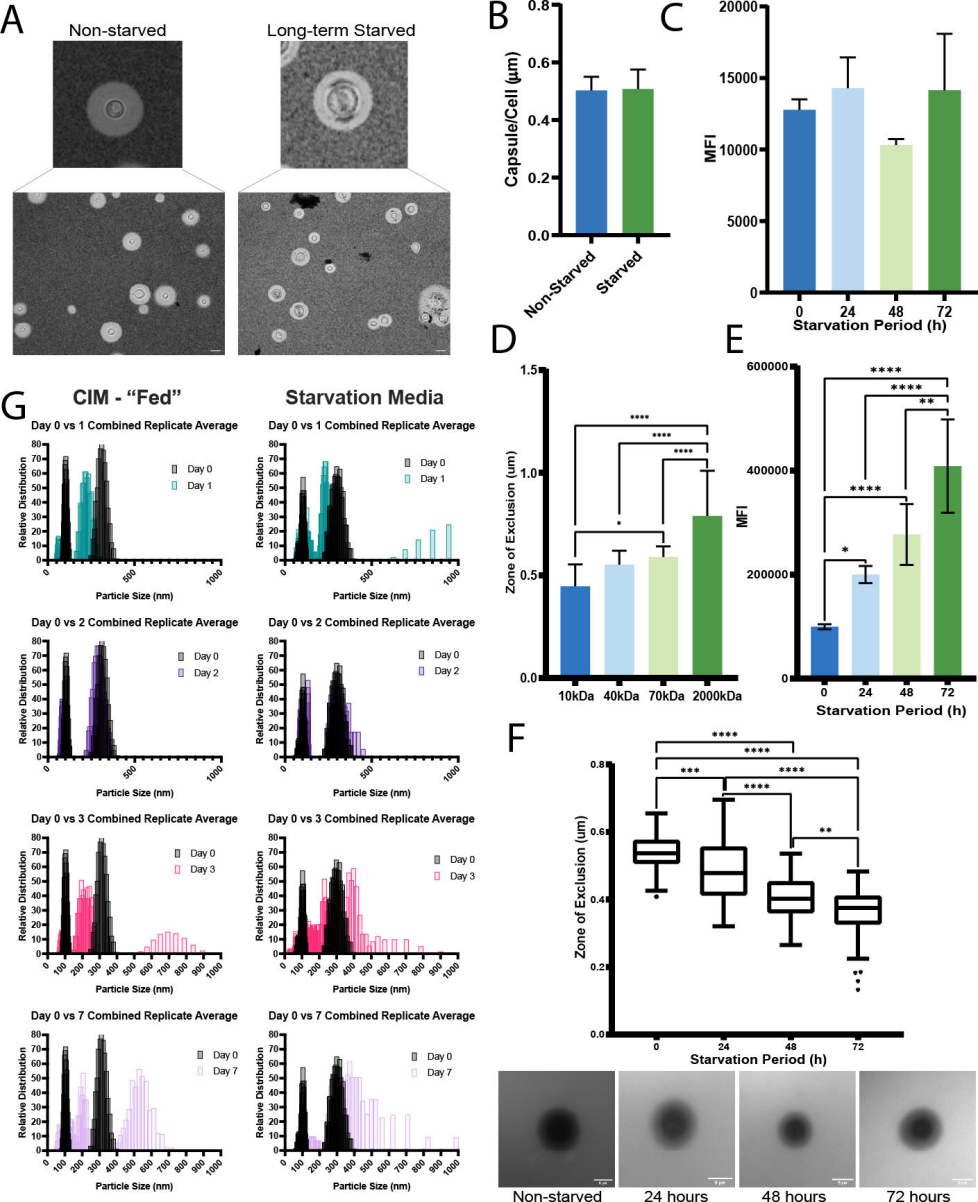# Erratum for Bedford et al., “Carbon starvation induces coincident capsule and cell wall remodeling in *Cryptococcus neoformans*”

**DOI:** 10.1128/mbio.00495-26

**Published:** 2026-03-23

**Authors:** Elise Bedford, Leandro Buffoni Roque da Silva, Daniel Smith, Christopher W. J. Lee, Quigly Dragotakes, Arturo Casadevall, James W. Kronstad

## ERRATUM

Volume 17, no. 2, e03701-25, 2025, https://doi.org/10.1128/mbio.03701-25. Figure 1: The panel locants (A to G) should appear as shown in this erratum.

**Fig 1:**