# Supplementary material for: Carbon starvation induces coincident capsule and cell wall remodeling in Cryptococcus neoformans
Source: mBio. 2025 Dec 30;17(2):e03701-25. doi: 10.1128/mbio.03701-25 (PMC12892975; doi:10.1128/mbio.03701-25)
Supplement: Fig. S1 — Measurements of capsule size, permeability, and antibody staining. [file mbio.03701-25-s0001.pdf]

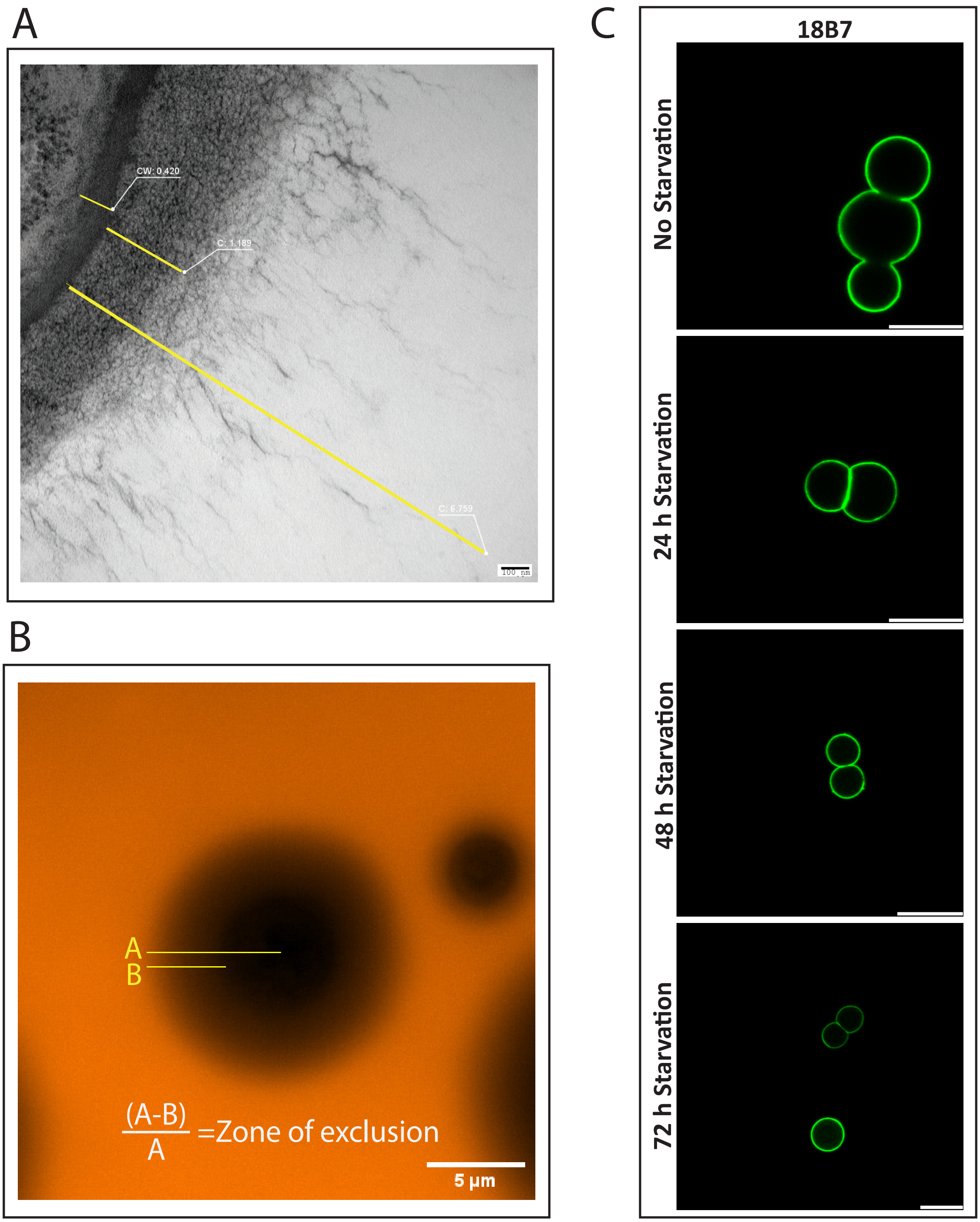

Figure S1. Measurements of capsule size, permeability and antibody staining. A. An example of a TEM image to illustrate the measurements taken in ImageJ. Yellow lines indicating the cell wall, the dense capsule layer and the full capsule. For the analysis, the pixel measurement was converted to µm using the magnification. B. Illustration of the zone of exclusion for testing dextran permeability. Measurements were taken in ImageJ with the first measurement A being the entire cell and the second measurement B being the permeated section of the cell. The zone of exclusion was calculated and normalized as the diameter of the non-permeated portion of the cell relative to cell size. C. Representative images for the mAb 18B7 staining for each time point. Images were taken using confocal microscopy and Leica software for analysis.
