# Supplementary material for: Carbon starvation induces coincident capsule and cell wall remodeling in Cryptococcus neoformans
Source: mBio. 2025 Dec 30;17(2):e03701-25. doi: 10.1128/mbio.03701-25 (PMC12892975; doi:10.1128/mbio.03701-25)
Supplement: Fig. S2 — The wild-type strain H99 is sensitive to cell wall stress upon starvation. [file mbio.03701-25-s0002.pdf]

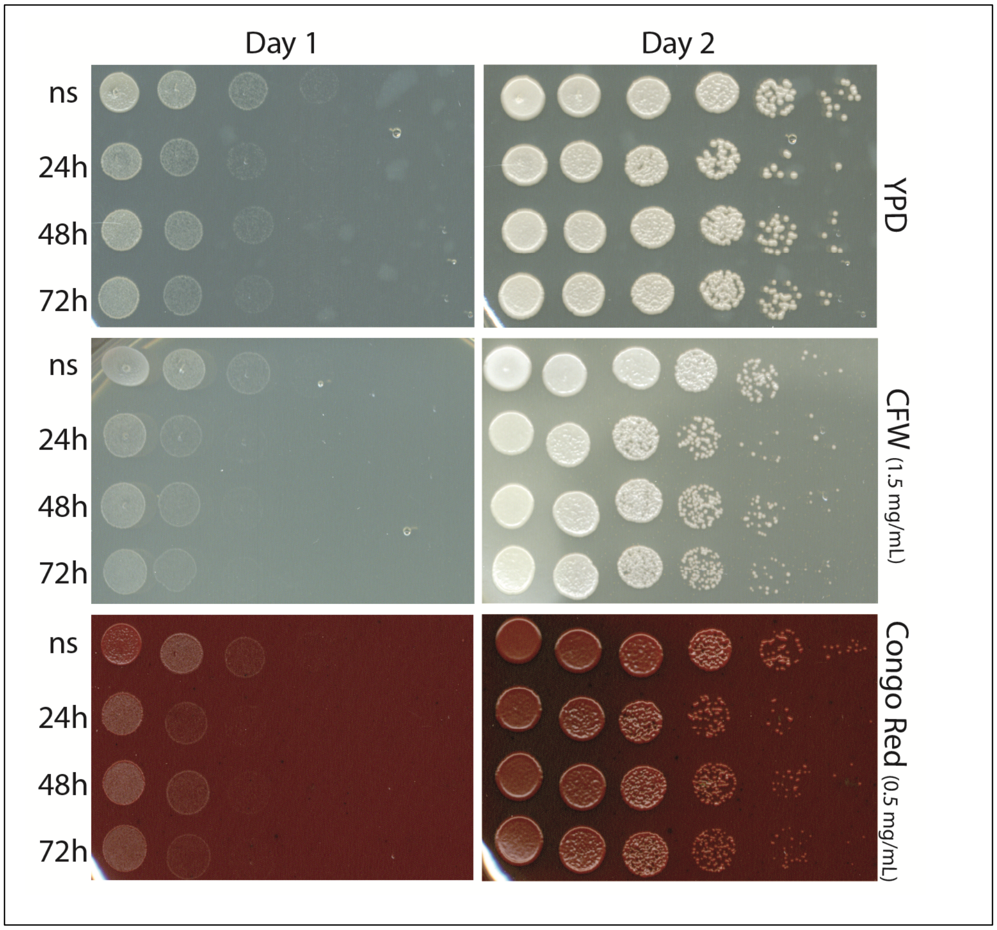

Figure S2. The wild-type strain H99 is sensitive to cell wall stress upon starvation. Spot assays were performed on YPD media without or with Congo red (0.5mg/mL) or CFW (1.5 mg/mL) to measure cell wall integrity of the designated starvation period after 24 and 48 h of growth at 30°C.
