# Supplementary material for: Carbon starvation induces coincident capsule and cell wall remodeling in Cryptococcus neoformans
Source: mBio. 2025 Dec 30;17(2):e03701-25. doi: 10.1128/mbio.03701-25 (PMC12892975; doi:10.1128/mbio.03701-25)
Supplement: Fig. S3 — Cell viability is not negatively impacted by carbon limitation. [file mbio.03701-25-s0003.pdf]

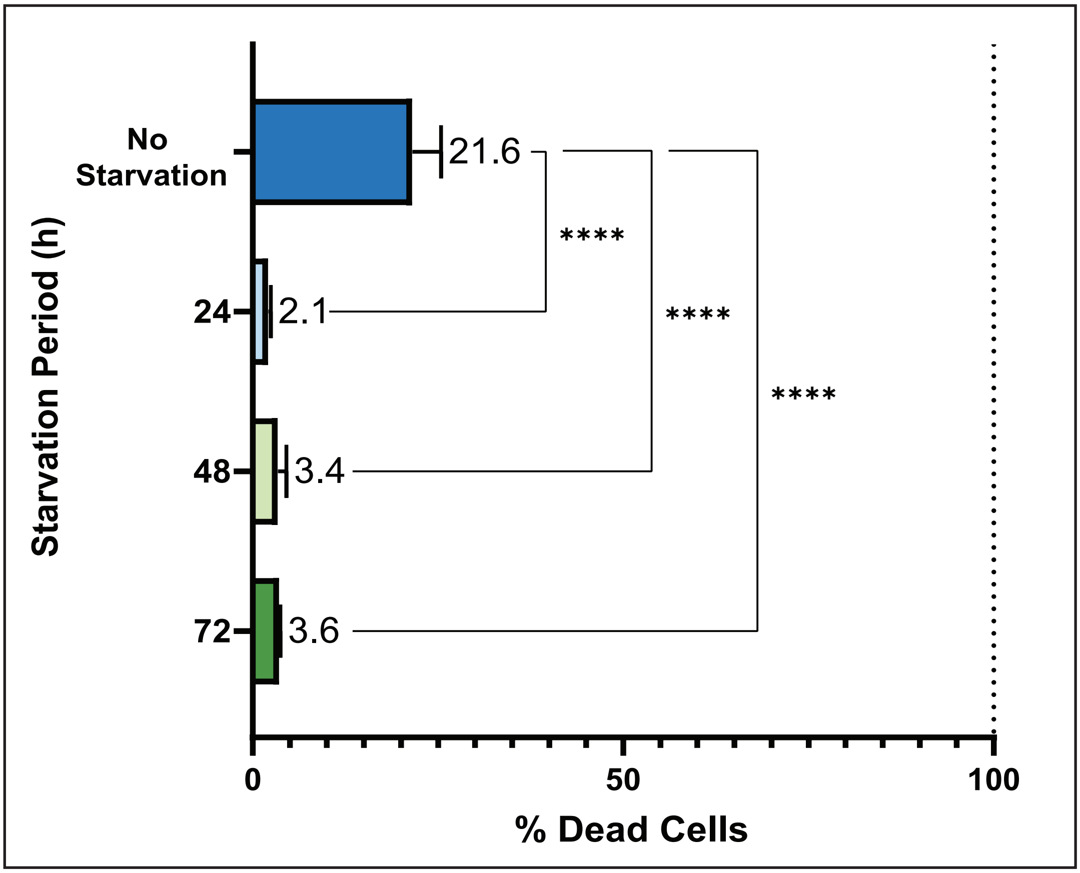

Figure S3. Cell viability is not negatively impacted by carbon limitation. Propidium iodide staining to distinguish live versus dead cells. The percentages of dead cells at each of the time points of starvation are indicated. Statistical significance was measured with an ordinary one-way ANOVA and the labeled significance was based on the results of the post-hoc comparisons if the p-value was statistically significant in the ANOVA summary (****$p<0.0001$).
