# Supplementary material for: Carbon starvation induces coincident capsule and cell wall remodeling in Cryptococcus neoformans
Source: mBio. 2025 Dec 30;17(2):e03701-25. doi: 10.1128/mbio.03701-25 (PMC12892975; doi:10.1128/mbio.03701-25)
Supplement: Fig. S4 — Starvation time impacts the rate of recovery of cells when reintroduced into a medium with glucose. [file mbio.03701-25-s0004.pdf]

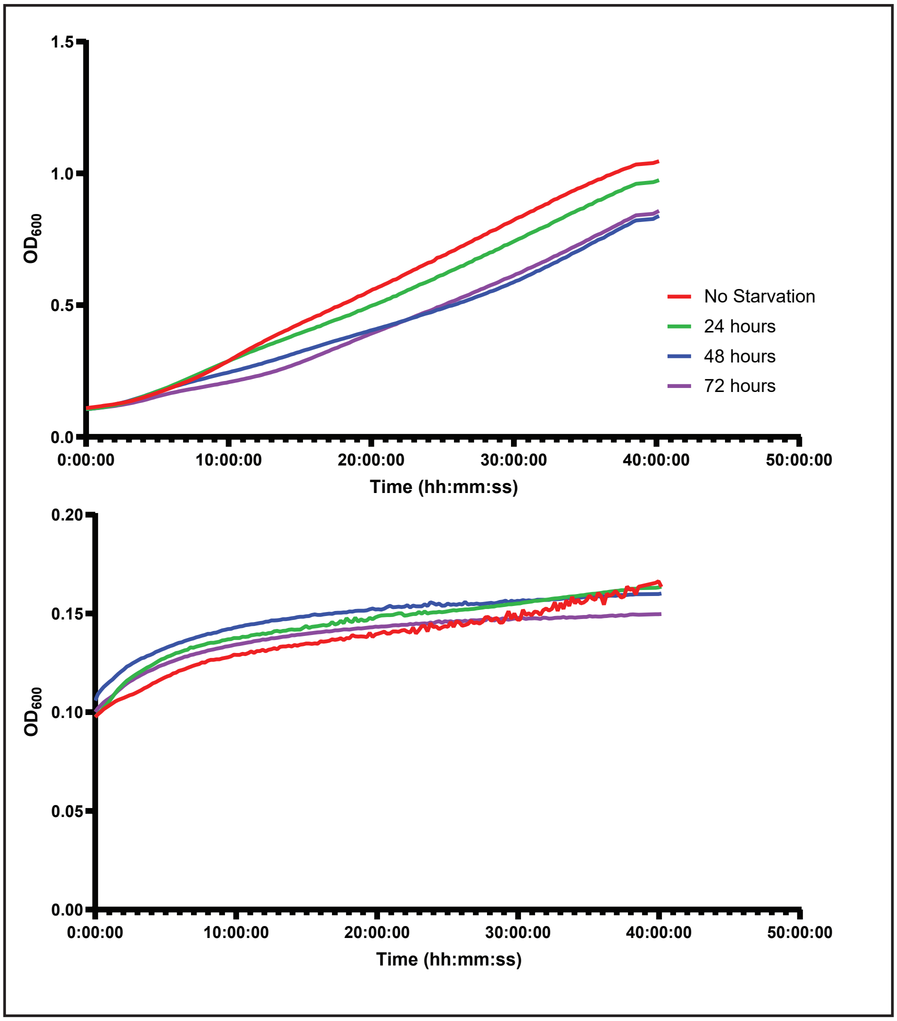

Figure S4. Starvation time impacts rate of recovery of cells when reintroduced into a medium with glucose. Both graphs illustrate the OD readings for each time point over a 40-hour period when cells were introduced into the medium with glucose, with the red line representing the non-starved samples, green line showing the 24-hour starvation, blue line showing the 48-hour starvation and the purple line showing the 72-hour starvation. The top graph shows the reintroduction into YPD (2% glucose) and the bottom graph shows the reintroduction into CIM (0.5% glucose).
