# Supplementary material for: Carbon starvation induces coincident capsule and cell wall remodeling in Cryptococcus neoformans
Source: mBio. 2025 Dec 30;17(2):e03701-25. doi: 10.1128/mbio.03701-25 (PMC12892975; doi:10.1128/mbio.03701-25)
Supplement: Fig. S5 — A higher percentage of starved cells are phagocytosed by a macrophage-like cell line compared to non-starved cells. [file mbio.03701-25-s0005.pdf]

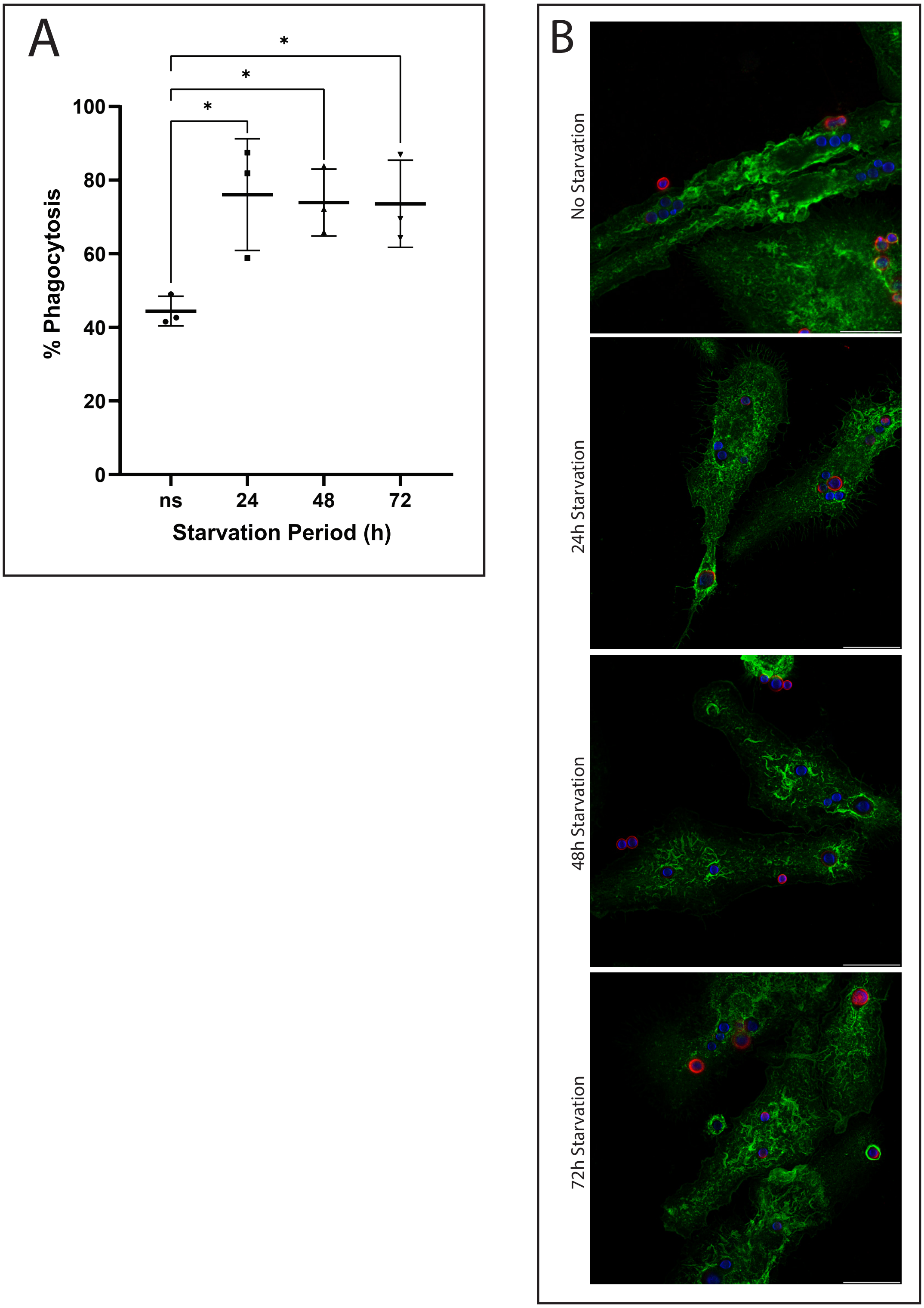

Figure S5. A higher percentage of starved cells are phagocytosed by a macrophage-like cell line compared to non-starved cells. A. The graph shows the percentage of macrophages phagocytosing cells from each of the conditions. The cells without starvation are designated ns. Statistical significance was measured with an ordinary one-way ANOVA and the labeled significance was based on the results of the post-hoc comparisons if the p-value was statistically significant in the ANOVA summary (*$p<0.05$). B. Representative images of fungal cell uptake by macrophages using confocal microscopy at each starvation period. The yeast cells are stained with calcofluor white (blue) to allow visualization. The 18b7 stained cells are indicated in red and this staining disappears once the yeast cells is phagocytosed. The actin fibrils of the macrophages are stained green.
