# Supplementary material for: Carbon starvation induces coincident capsule and cell wall remodeling in Cryptococcus neoformans
Source: mBio. 2025 Dec 30;17(2):e03701-25. doi: 10.1128/mbio.03701-25 (PMC12892975; doi:10.1128/mbio.03701-25)
Supplement: Fig. S6 — Heatmap of the RNA-seq data demonstrating differential expression of specific genes in the glucose and starvation conditions. [file mbio.03701-25-s0006.pdf]

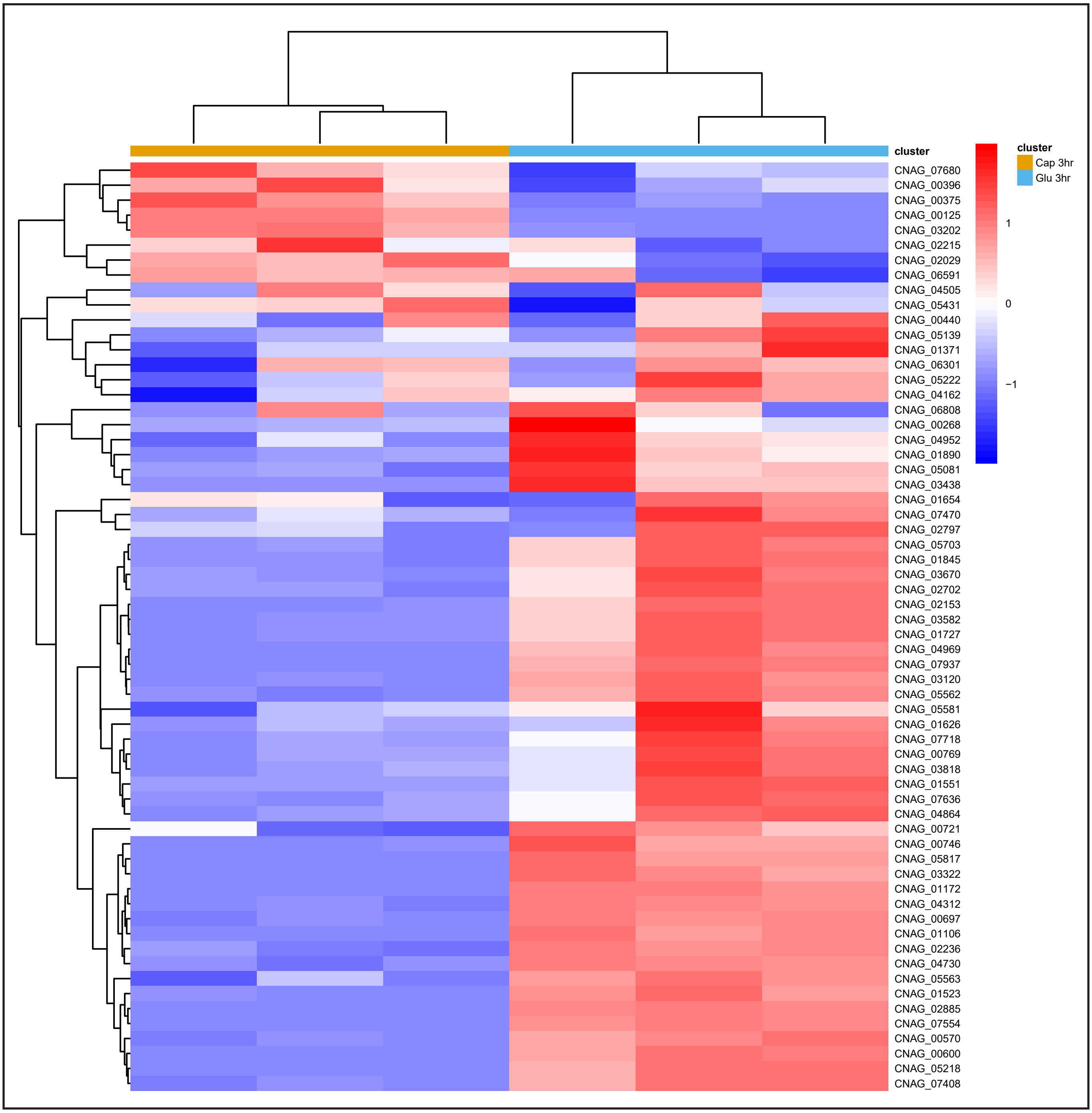

Figure S6. Heatmap of the RNA-seq data demonstrating differential expression of specific genes in the glucose and starvation conditions. A heatmap is shown for the known capsule- related genes in *C. neoformans*. The orange column shows the expression of the designated genes in the starvation condition and the blue column shows the expression of the designated genes in the glucose condition.
