# Supplementary material for: Carbon starvation induces coincident capsule and cell wall remodeling in Cryptococcus neoformans
Source: mBio. 2025 Dec 30;17(2):e03701-25. doi: 10.1128/mbio.03701-25 (PMC12892975; doi:10.1128/mbio.03701-25)
Supplement: Fig. S7 — Heatmap of the RNA-seq data demonstrating differential expression of specific genes in the glucose and starvation conditions. [file mbio.03701-25-s0007.pdf]

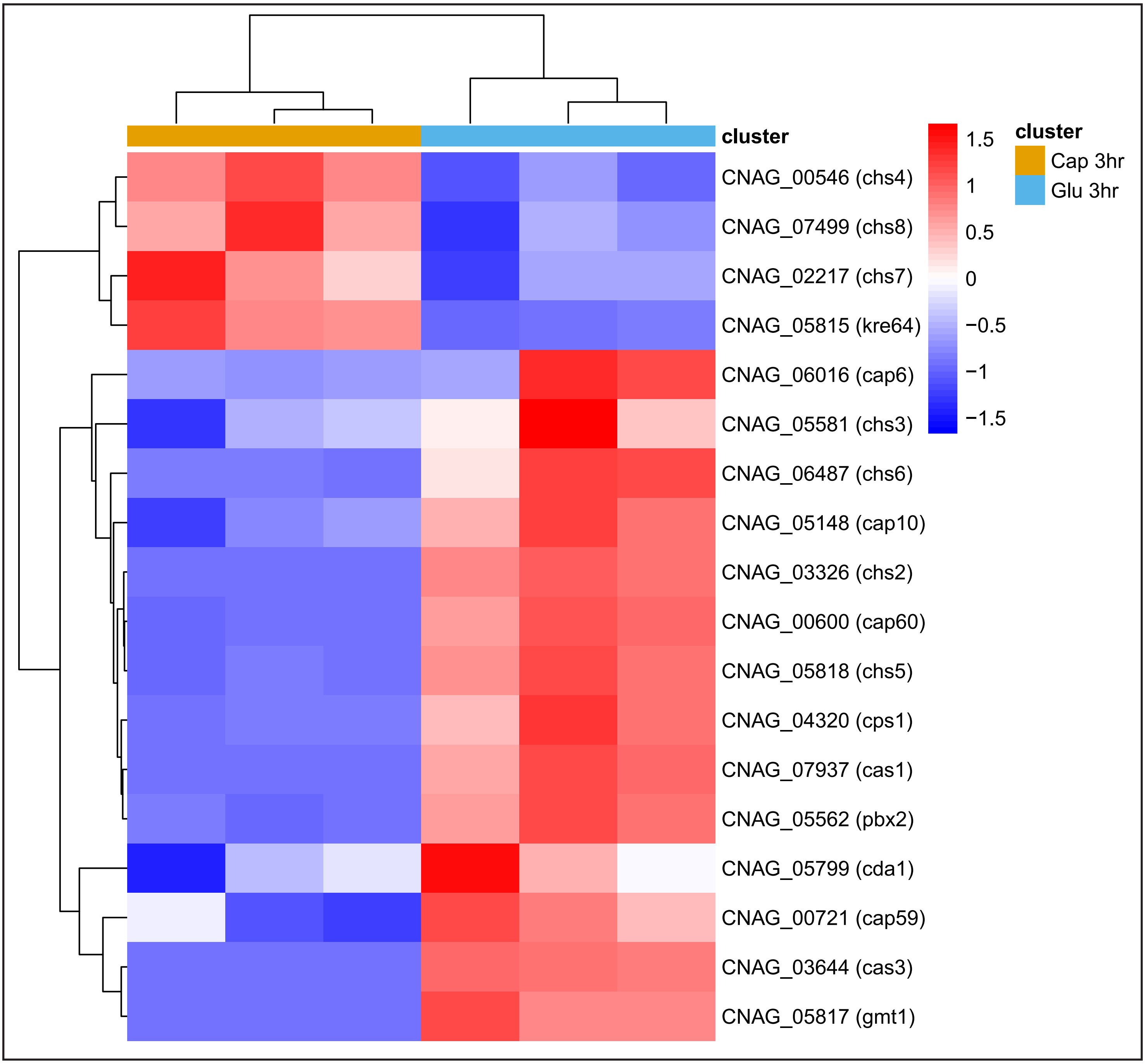

Figure S7. Heatmap of the RNA-seq data demonstrating differential expression of specific genes in the glucose and starvation conditions. The heatmap depicts the genes that were analyzed further in this study. The orange column shows the expression of the designated genes in the starvation condition and the blue column shows the expression of the designated genes in the glucose condition.
