# Supplementary material for: Carbon starvation induces coincident capsule and cell wall remodeling in Cryptococcus neoformans
Source: mBio. 2025 Dec 30;17(2):e03701-25. doi: 10.1128/mbio.03701-25 (PMC12892975; doi:10.1128/mbio.03701-25)
Supplement: Fig. S8 — Summary of the RNA-seq data illustrating the difference in gene expression between the glucose and starvation conditions. [file mbio.03701-25-s0008.pdf]

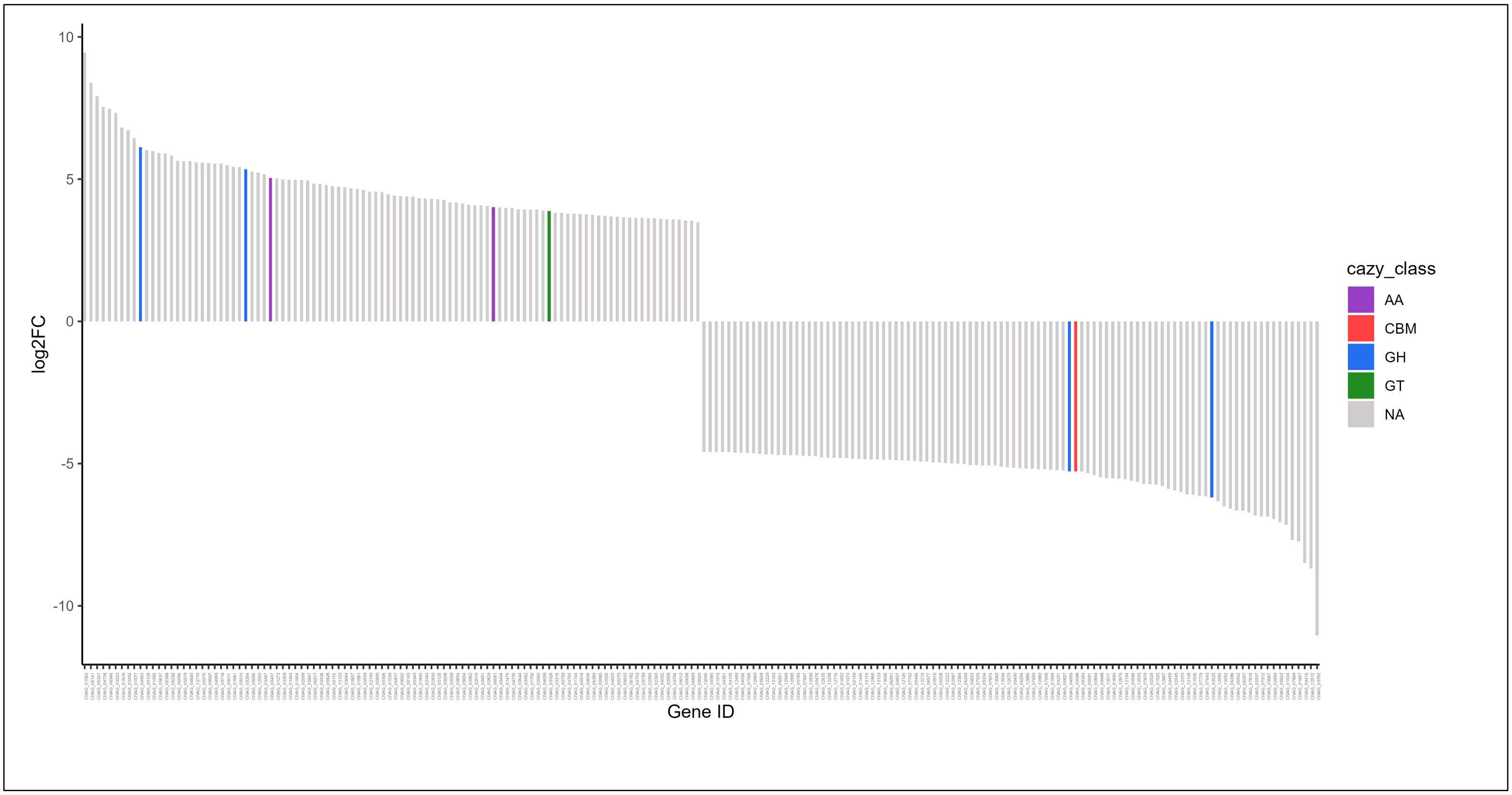

Figure S8. Summary of the RNA-seq data illustrating the difference in gene expression between the glucose and starvation conditions. Histogram of the top 100 differentially expressed genes in both conditions with the carbohydrate active enzymes highlighted based on class. The bars on the left are the glucose condition and the bars on the right are the starvation condition. Lists of the genes in histogram can be found in tables S1 and S2.
