# Supplementary material for: Carbon starvation induces coincident capsule and cell wall remodeling in Cryptococcus neoformans
Source: mBio. 2025 Dec 30;17(2):e03701-25. doi: 10.1128/mbio.03701-25 (PMC12892975; doi:10.1128/mbio.03701-25)
Supplement: Fig. S9 — Representative images of the zone of exclusion for dextran permeability with the acapsular mutants. [file mbio.03701-25-s0009.pdf]

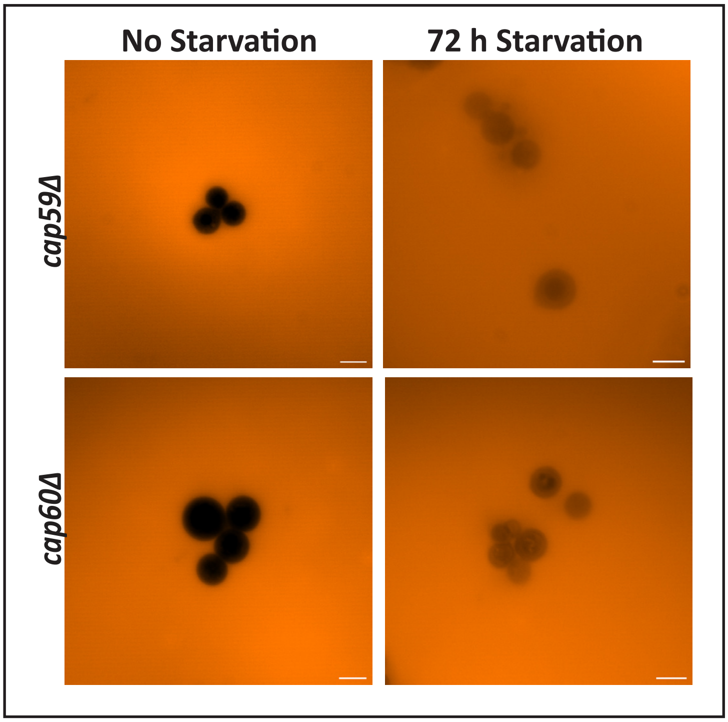

Figure S9. Representative images of the zone of exclusion for dextran permeability with the acapsular mutants. The cells were imaged and measurements were taken in ImageJ, where full cell permeation of the dextran could be visualized. Images were acquired at the same exposure time and normalized to the same intensity value. Bar = 5 microns
