# Supplementary material for: Carbon starvation induces coincident capsule and cell wall remodeling in Cryptococcus neoformans
Source: mBio. 2025 Dec 30;17(2):e03701-25. doi: 10.1128/mbio.03701-25 (PMC12892975; doi:10.1128/mbio.03701-25)
Supplement: Supplemental Tables — Tables S1 to S3. [file mbio.03701-25-s0010.pdf]

Table S1. Top 100 upregulated genes from the RNA-seq data in the glucose condition.

Gene ID	Product Description	log2FoldChange*	lfcSE*	stat*	pvalue*
CNAG_00005	TPR repeat-containing protein	4.137693	2.792095	1.481932	0.138359
CNAG_00085	histone chaperone ASF1	3.718592	0.33502	11.09961	1.26E-28
CNAG_00099	minichromosome maintenance protein 3	4.184777	0.265909	15.73766	8.35E-56
CNAG_00110	hypothetical protein	4.749308	0.467059	10.16854	2.74E-24
CNAG_00128	hypothetical protein	6.020915	0.669929	8.987394	2.53E-19
CNAG_00165	methylthioadenosine phosphorylase	4.396567	0.305706	14.3817	6.74E-47
CNAG_00275	hypothetical protein	3.684303	0.313464	11.75353	6.77E-32
CNAG_00306	copper-detoxifying metallothionein 2	4.54363	0.269441	16.86317	8.40E-64
CNAG_00349	hypothetical protein	4.378002	0.471958	9.276249	1.76E-20
CNAG_00407	glyoxal oxidase	5.041172	0.224206	22.48454	5.88E-112
CNAG_00451	cytoplasmic protein	4.074254	0.208117	19.57673	2.44E-85
CNAG_00454	hypothetical protein	4.001997	0.222854	17.95793	4.16E-72
CNAG_00539	membrane transporter	4.618935	0.300124	15.3901	1.91E-53
CNAG_00709	uracil-DNA glycosylase	3.812313	0.177416	21.48798	2.02E-102
CNAG_00854	C-8 sterol isomerase	4.175482	0.205209	20.34747	4.89E-92
CNAG_01272	hypothetical protein	5.019917	0.424406	11.82809	2.79E-32
CNAG_01464	flavohemoglobin	4.980477	0.283415	17.57308	3.96E-69

CNAG_01475	sphingolipid C9-methyltransferase	3.99575	0.161031	24.81361	6.39E-136
CNAG_01539	myo-inositol-1-phosphate synthase	4.461658	0.170116	26.22717	1.30E-151
CNAG_01562	pr4/barwin domain protein	9.44505	0.161923	58.33034	0
CNAG_01577	glutamate dehydrogenase (NADP)	6.441521	0.185048	34.81007	1.71E-265
CNAG_01664	CMGC/CDK/CDC2 protein kinase	4.974897	0.226925	21.92304	1.57E-106
CNAG_01681	cytosine permease	5.431964	0.405558	13.39379	6.57E-41
CNAG_01744	phosphatase	3.784179	0.146018	25.91592	4.41E-148
CNAG_01816	hypothetical protein, hypothetical protein, variant	6.814954	0.601754	11.32515	9.85E-30
CNAG_01840	tubulin beta chain	4.325529	0.120429	35.91764	1.62E-282
CNAG_01981	sulfide:quinone oxidoreductase	4.649967	0.511869	9.084287	1.04E-19
CNAG_01995	hypothetical protein	5.989375	1.671049	3.584201	0.000338
CNAG_02010	tartrate transporter, variant, tartrate transporter	4.076595	0.231598	17.60203	2.38E-69
CNAG_02060	hypothetical protein	3.926408	0.220674	17.79276	8.04E-71
CNAG_02099	fatty acid synthase subunit beta, fungi type	4.966012	0.202557	24.51658	9.83E-133
CNAG_02100	fatty acid synthase subunit alpha, fungi type	4.561122	0.218888	20.83773	1.97E-96
CNAG_02343	hypothetical protein	4.322402	0.231408	18.67872	7.38E-78

CNAG_02777	phosphate:H symporter, variant, phosphate:H symporter	3.767048	1.22714	3.069778	NA
CNAG_02829	protein arginine N-methyltransferase 5	4.057261	0.275353	14.73474	3.86E-49
CNAG_02830	delta24(24(1))-sterol reductase	4.307546	0.163617	26.32699	9.42E-153
CNAG_02935	malonic semialdehyde reductase	3.713788	0.203129	18.28291	1.13E-74
CNAG_02990	nuclear protein	3.629274	0.145767	24.89771	7.88E-137
CNAG_03044	hypothetical protein, variant, hypothetical protein	4.721728	0.333247	14.16887	1.43E-45
CNAG_03125	hypothetical protein	4.295353	0.76738	5.597427	2.18E-08
CNAG_03223	hypothetical protein	7.328589	0.186804	39.23152	0
CNAG_03316	rho gdp-dissociation inhibitor	3.816805	0.201497	18.94226	5.12E-80
CNAG_03326	chitin synthase	3.874332	0.211853	18.28781	1.03E-74
CNAG_03369	WEE protein kinase	4.995219	1.758513	2.840592	0.004503
CNAG_03438	hexose transporter	4.831313	0.302125	15.99113	1.47E-57
CNAG_03453	kinesin family member 11	6.718922	0.900341	7.46264	8.48E-14
CNAG_03482	Thiol peroxidase	3.929505	0.184226	21.32981	6.01E-101
CNAG_03487	DnaJ domain-containing protein	5.172429	0.273536	18.90947	9.53E-80
CNAG_03644	capsule related protein	3.94096	0.152628	25.82073	5.19E-147
CNAG_03716	pr4/barwin domain protein	5.542303	0.185179	29.92947	8.14E-197
CNAG_03857	hypothetical protein	4.679171	0.223509	20.935	2.57E-97

CNAG_03962	minichromosome maintenance protein 6	4.102218	0.298692	13.73392	6.36E-43
CNAG_04603	replication fork protection complex subunit Csm3/Swi3	3.693235	0.261089	14.1455	1.99E-45
CNAG_04635	endopeptidase	3.762955	0.310307	12.12654	7.64E-34
CNAG_04640	ATP-citrate synthase subunit 1	5.626567	0.17175	32.7602	2.17E-235
CNAG_04659	pyruvate decarboxylase	3.895181	0.165102	23.59263	4.59E-123
CNAG_04735	extracellular elastinolytic metalloproteinase	3.985654	0.385069	10.35049	4.16E-25
CNAG_04758	amt family ammonium transporter	7.540483	1.241204	6.075137	1.24E-09
CNAG_04760	cytoplasmic protein	3.788758	0.183212	20.67965	5.28E-95
CNAG_04793	hypothetical protein	3.64194	0.405953	8.971342	2.93E-19
CNAG_04963	hypothetical protein, variant, hypothetical protein	6.121818	0.163724	37.39103	5.47E-306
CNAG_04969	UDP-glucose 6-dehydrogenase	5.545568	0.197105	28.13513	3.64E-174
CNAG_05079	hypothetical protein	5.632343	0.683059	8.245765	1.64E-16
CNAG_05264	alpha-amylase AmyA, alpha-amylase AmyA, variant	5.35013	0.28433	18.81664	5.52E-79
CNAG_05429	hypothetical protein	4.515445	2.509169	1.799578	0.071927
CNAG_05576	hypothetical protein	5.581201	0.475883	11.7281	9.15E-32
CNAG_05637	hypothetical protein	5.925416	0.323262	18.33009	4.76E-75

CNAG_05759	acetyl-CoA carboxylase/biotin carboxylase	3.638658	0.224539	16.20502	4.65E-59
CNAG_05828	UDP-N-acetylglucosamine pyrophosphorylase	4.79092	0.263185	18.20364	4.83E-74
CNAG_05847	thioredoxin reductase	4.955984	0.184808	26.81693	2.05E-158
CNAG_06081	glucose oxidase	4.011283	0.227989	17.59418	2.73E-69
CNAG_06096	tricarboxylate carrier	5.639609	0.136182	41.4124	0
CNAG_06141	dUTP pyrophosphatase	8.38331	0.564938	14.83934	8.16E-50
CNAG_06182	minichromosome maintenance protein 4 (cell division control protein 54)	3.64661	0.221167	16.48801	4.47E-61
CNAG_06256	hypothetical protein	5.828446	1.462686	3.984757	6.75E-05
CNAG_06346	barwin-like protein 1	7.471495	0.235923	31.66925	4.12E-220
CNAG_06347	pr4/barwin domain protein	7.917635	0.32554	24.32151	1.16E-130
CNAG_06388	hypothetical protein	5.90261	0.329763	17.89953	1.19E-71
CNAG_06389	hypothetical protein	3.749872	0.360683	10.39658	2.57E-25
CNAG_06817	uric acid xanthine permease	4.427992	0.254452	17.40209	7.96E-68
CNAG_06890	membrane transporter	4.560895	0.107526	42.41664	0
CNAG_06914	tubulin gamma chain	3.771544	0.197737	19.07356	4.19E-81
CNAG_06917	thiol-specific antioxidant protein 3	4.841029	0.449882	10.76066	5.28E-27
CNAG_07022	hypothetical protein	3.745346	2.820095	1.328092	0.184148
CNAG_07756	cell division control protein	3.926891	0.620145	6.332209	2.42E-10

CNAG_09001	hypothetical protein	3.962998	1.616029	2.452307	0.014194
CNAG_09002	apocytochrome b	4.399555	0.842608	5.221355	1.78E-07
CNAG_09003	small ribosomal protein subunit 3	3.654601	1.337008	2.733418	0.006268
CNAG_09004	cytochrome c oxidase subunit III	4.141271	0.567251	7.3006	2.86E-13
CNAG_09006	NADH:ubiquinone oxidoreductase subunit 5	5.26681	0.924	5.700011	1.20E-08
CNAG_09007	NADH dehydrogenase subunit 6	5.56315	1.516721	3.667879	0.000245
CNAG_09008	ATP synthase subunit 6	4.266951	1.114753	3.82771	0.000129
CNAG_09010	NADH dehydrogenase subunit 2	5.427318	1.315131	4.126826	3.68E-05
CNAG_09011	NADH dehydrogenase subunit 3	5.490827	1.61772	3.394176	0.000688
CNAG_12206	unspecified product	3.649356	1.520774	2.399671	0.01641
CNAG_12262	unspecified product	4.507007	2.020772	2.230339	0.025725
CNAG_12560	unspecified product	5.227944	1.623732	3.219709	0.001283
CNAG_12770	unspecified product	5.58589	0.204118	27.36595	6.98E-165
CNAG_12950	unspecified product	4.755737	1.998105	2.380124	0.017306831
CNAG_13120	unspecified product	4.733088	0.223058	21.2191	6.36E-100

*The standard error estimate (lfcSE), test statistic (stat) and p-value are shown with the log2 fold change for the transcripts analyzed by DESeq2 (Love MI, Huber W, Anders S. Moderated estimation of fold change and dispersion for RNA-seq data with DESeq2. *Genome Biol*. 2014;15(12):550. doi:10.1186/s13059-014-0550-8).

Table S2. Top 100 upregulated genes from the RNA-seq data in the starvation condition.

Gene ID	Product Description	log2FoldChange*	lfcSE*	stat*	pvalue*
CNAG_00091	hypothetical protein	-5.33756	0.3607	-14.7978	1.51E-49
CNAG_00476	hypothetical protein	-4.74125	0.420128	-11.2853	1.55E-29
CNAG_00488	hypothetical protein, hypothetical protein, variant	-5.47579	0.46502	-11.7754	5.23E-32
CNAG_00490	acetyl-CoA acyltransferase	-5.14486	0.196611	-26.1677	6.20E-151
CNAG_00588	hypothetical protein	-5.27474	0.173513	-30.3996	5.55E-203
CNAG_00598	nicotinamide mononucleotide permease	-5.59911	0.159524	-35.0988	7.03E-270
CNAG_00827	ribose 5-phosphate isomerase	-5.24053	0.209954	-24.9604	1.65E-137
CNAG_00844	hypothetical protein	-5.40599	0.458689	-11.7857	4.62E-32
CNAG_01052	hypothetical protein	-4.79909	0.177412	-27.0506	3.72E-161
CNAG_01070	class II aldolase/adducin family protein	-4.80457	0.224737	-21.3786	2.11E-101
CNAG_01446	heat shock protein, 12Kda	-4.84442	0.349149	-13.8749	8.99E-44
CNAG_01534	hypothetical protein	-7.15077	0.147793	-48.3836	0
CNAG_01690	MFS transporter, variant 1, MFS transporter, MFS transporter, variant 2	-5.51152	0.227829	-24.1915	2.73E-129
CNAG_01847	hypothetical protein	-7.73543	0.232498	-33.271	1.01E-242
CNAG_01919	hypothetical protein	-4.59421	0.190019	-24.1776	3.83E-129
CNAG_01925	hypothetical protein	-5.73894	0.593082	-9.67648	3.80E-22

CNAG_01936	sugar transporter	-6.09262	0.283565	-21.4858	2.11E-102
CNAG_01994	hypothetical protein	-5.20765	0.258615	-20.1367	3.52E-90
CNAG_02045	acetoacetate-CoA ligase	-5.95016	0.28704	-20.7294	1.88E-95
CNAG_02169	hypothetical protein	-4.83615	0.178731	-27.0583	3.05E-161
CNAG_02526	hypothetical protein	-5.72023	0.307921	-18.577	4.93E-77
CNAG_02537	hypothetical protein	-6.82245	1.078039	-6.32858	2.47E-10
CNAG_02899	hypothetical protein	-6.93723	0.255411	-27.161	1.88E-162
CNAG_02987	hypothetical protein	-4.99287	0.748346	-6.67188	2.53E-11
CNAG_03067	hydroxymethylglutaryl-CoA lyase	-6.86045	0.250817	-27.3524	1.01E-164
CNAG_03525	alpha,alpha-trehalase	-6.19166	1.052379	-5.88349	4.02E-09
CNAG_03782	hypothetical protein	-11.0408	0.372429	-29.6454	3.89E-193
CNAG_03783	hypothetical protein	-4.71048	0.223143	-21.1097	6.48E-99
CNAG_03910	myo-inositol transporter, putative	-4.95876	0.128416	-38.6149	0
CNAG_04105	hypothetical protein	-4.59754	0.329923	-13.9352	3.87E-44
CNAG_04201	hypothetical protein	-5.22989	0.306941	-17.0388	4.24E-65
CNAG_04351	methylmalonate-semialdehyde dehydrogenase (acylating)	-4.59514	0.337735	-13.6057	3.70E-42
CNAG_04416	major facilitator superfamily transporter	-8.49767	0.321026	-26.4703	2.13E-154
CNAG_04459	hypothetical protein	-5.88184	1.392716	-4.22328	2.41E-05

CNAG_04536	nicotinamide mononucleotide permease, variant, nicotinamide mononucleotide permease	-4.61834	0.332957	-13.8707	9.54E-44
CNAG_04585	hypothetical protein	-4.59184	0.226721	-20.2532	3.33E-91
CNAG_04623	hypothetical protein	-5.05497	0.375709	-13.4545	2.90E-41
CNAG_04837	bHLH family transcription factor	-4.88339	0.276172	-17.6824	5.73E-70
CNAG_04993	hypothetical protein	-5.27395	1.831586	-2.87944	0.003984
CNAG_05303	isocitrate lyase	-5.27709	0.264837	-19.9258	2.43E-88
CNAG_05334	hypothetical protein, variant, hypothetical protein	-5.05999	0.392712	-12.8847	5.49E-38
CNAG_05466	hypothetical protein	-4.91189	1.579511	-3.10975	0.001872
CNAG_05528	hypothetical protein	-4.96203	0.231007	-21.48	2.40E-102
CNAG_05659	hypothetical protein	-4.65194	0.589407	-7.89257	2.96E-15
CNAG_05662	polyol transporter protein 1	-7.05382	0.364429	-19.3558	1.82E-83
CNAG_06209	hypothetical protein	-5.01128	0.415283	-12.0672	1.57E-33
CNAG_06267	hypothetical protein	-6.66082	0.149167	-44.6534	0
CNAG_06294	hypothetical protein	-5.15895	0.372922	-13.8339	1.59E-43
CNAG_06396	hypothetical protein	-6.58123	0.486593	-13.5251	1.11E-41
CNAG_06551	carnitine O-acetyltransferase	-4.86909	0.318861	-15.2703	1.21E-52
CNAG_06561	allantoate transporter	-4.69647	0.352024	-13.3413	1.33E-40
CNAG_06577	hypothetical protein	-4.93059	0.315219	-15.6418	3.78E-55

CNAG_06932	sugar transporter	-6.65307	0.311228	-21.3768	2.20E-101
CNAG_07008	hypothetical protein, variant, hypothetical protein	-5.20145	1.631064	-3.189	0.001428
CNAG_07505	hypothetical protein, variant, hypothetical protein	-4.62185	0.35978	-12.8463	9.02E-38
CNAG_07525	hypothetical protein	-5.05841	0.507052	-9.97611	1.94E-23
CNAG_07643	hypothetical protein	-6.1602	1.417381	-4.34618	1.39E-05
CNAG_07693	high-affinity methionine permease, variant, high-affinity methionine permease	-5.18469	0.918225	-5.64642	1.64E-08
CNAG_07742	hypothetical protein	-4.90095	0.991264	-4.94414	7.65E-07
CNAG_07779	D-glycerate 3-kinase	-6.13283	0.513233	-11.9494	6.54E-33
CNAG_07816	2-hydroxyacid dehydrogenase	-6.72079	1.256039	-5.35079	8.76E-08
CNAG_07847	hypothetical protein	-4.72125	0.984575	-4.79522	1.62E-06
CNAG_07869	hypothetical protein	-7.68443	0.268611	-28.608	5.34E-180
CNAG_07870	hypothetical protein	-5.71076	0.802176	-7.11909	1.09E-12
CNAG_07874	sugar transporter	-5.06057	0.619489	-8.16895	3.11E-16
CNAG_07912	hypothetical protein	-6.85571	0.462483	-14.8237	1.03E-49
CNAG_12054	unspecified product	-6.32122	1.296279	-4.87643	1.08E-06
CNAG_12066	unspecified product	-4.58597	0.363844	-12.6042	2.00E-36
CNAG_12123	unspecified product	-4.85712	1.62649	-2.98626	0.002824

CNAG_12126	unspecified product	-4.8894	1.517884	-3.22119	0.001277
CNAG_12128	unspecified product	-6.07694	1.36673	-4.44634	8.73E-06
CNAG_12154	unspecified product	-5.54371	1.47677	-3.75395	0.000174
CNAG_12208	unspecified product	-4.78954	0.462469	-10.3565	3.91E-25
CNAG_12210	unspecified product	-4.92557	1.61304	-3.0536	0.002261
CNAG_12222	unspecified product	-4.98228	0.454442	-10.9635	5.72E-28
CNAG_12229	unspecified product	-4.67315	0.3414	-13.6882	1.19E-42
CNAG_12354	unspecified product	-4.99957	0.754143	-6.62947	3.37E-11
CNAG_12370	unspecified product	-6.00142	0.326979	-18.3541	3.06E-75
CNAG_12485	unspecified product	-4.61045	0.315783	-14.6001	2.81E-48
CNAG_12512	unspecified product	-8.67846	0.85186	-10.1877	2.25E-24
CNAG_12533	unspecified product	-5.64619	0.791553	-7.13306	9.82E-13
CNAG_12635	unspecified product	-4.77753	1.605331	-2.97604	0.00292
CNAG_12675	unspecified product	-5.12731	0.422547	-12.1343	6.95E-34
CNAG_12716	unspecified product	-4.78973	1.647356	-2.90752	0.003643
CNAG_12847	unspecified product	-5.78949	1.454307	-3.98092	6.86E-05
CNAG_12870	unspecified product	-5.54138	0.407771	-13.5895	4.63E-42
CNAG_12885	unspecified product	-5.16495	1.535677	-3.36331	0.00077
CNAG_12929	unspecified product	-4.69937	1.643753	-2.85892	0.004251
CNAG_12936	unspecified product	-4.72543	1.189114	-3.97391	7.07E-05

CNAG_12966	unspecified product	-4.85569	1.580375	-3.07249	0.002123
CNAG_12969	unspecified product	-4.63576	0.375555	-12.3438	5.26E-35
CNAG_12989	unspecified product	-5.19936	0.680163	-7.64428	2.10E-14
CNAG_12995	unspecified product	-4.70947	1.394125	-3.37808	0.00073
CNAG_13011	unspecified product	-5.50652	1.472666	-3.73915	0.000185
CNAG_13026	unspecified product	-4.86095	0.433832	-11.2047	3.87E-29
CNAG_13034	unspecified product	-5.10374	0.42997	-11.87	1.70E-32
CNAG_13052	unspecified product	-6.50394	1.310038	-4.96469	6.88E-07
CNAG_13082	unspecified product	-5.06599	0.538401	-9.40932	4.99E-21
CNAG_13119	unspecified product	-4.84533	1.696289	-2.85643	0.004284
CNAG_13162	unspecified product	-4.67383	0.271459	-17.2175	1.96E-66

*The standard error estimate (lfcSE), test statistic (stat) and p-value are shown with the log2 fold change for the transcripts analyzed by DESeq2 (Love MI, Huber W, Anders S. Moderated estimation of fold change and dispersion for RNA-seq data with DESeq2. *Genome Biol*. 2014;15(12):550. doi:10.1186/s13059-014-0550-8).

Table S3. Strain List. The collection of *C. neoformans* mutants from the Madhani deletion collection were obtained from the Fungal Genetics Stock Center, and are in the KN99α background. The H99 strain is the parent of the deletion mutants other than those from the Madhani Knockout Collection. The gene number is provided for each of the mutants.

**Strain**	**Knockout Collection Year**	**Gene**
C. neoformans: KN99α *pbx2Δ::NAT*	2016	CNAG_05562
C. neoformans: KN99α *kre64Δ::NAT*	2016	CNAG_05815
C. neoformans: KN99α *cap6Δ::NAT*	2016	CNAG_06016
C. neoformans: KN99α *cas1Δ::NAT*	2015	CNAG_07937
chs1Δ	N/A	CNAG_03099
chs2Δ	N/A	CNAG_03326
chs3Δ	N/A	CNAG_05581
chs4Δ	N/A	CNAG_00546
chs5Δ	N/A	CNAG_05818
chs6Δ	N/A	CNAG_06487
chs7Δ	N/A	CNAG_02217
chs8Δ	N/A	CNAG_07499
cda1Δ	N/A	CNAG_05799
cap59Δ	N/A	CNAG_00721
cap60Δ	N/A	CNAG_00600
cps1Δ	N/A	CNAG_04320
cas3Δ	N/A	CNAG_03644
